# Kinematic Modeling of a Combined System of Multiple Mecanum-Wheeled Robots with Velocity Compensation

**DOI:** 10.3390/s20010075

**Published:** 2019-12-21

**Authors:** Yunwang Li, Shirong Ge, Sumei Dai, Lala Zhao, Xucong Yan, Yuwei Zheng, Yong Shi

**Affiliations:** 1 School of Mechatronic Engineering, China University of Mining and Technology, Xuzhou 221116, China; 2 Department of Mechanical Engineering, Stevens Institute of Technology, Hoboken, NJ 07030, USA; 3 School of Mechanical and Electrical Engineering, Xuzhou University of Technology, Xuzhou 221018, China

**Keywords:** kinematic model, robot combination system, Mecanum-wheeled robot, velocity compensation, cooperative motion

## Abstract

In industry, combination configurations composed of multiple Mecanum-wheeled mobile robots are adopted to transport large-scale objects. In this paper, a kinematic model with velocity compensation of the combined mobile system is created, aimed to provide a theoretical kinematic basis for accurate motion control. Motion simulations of a single four-Mecanum-wheeled virtual robot prototype on RecurDyn and motion tests of a robot physical prototype are carried out, and the motions of a variety of combined mobile configurations are also simulated. Motion simulation and test results prove that the kinematic models of single- and multiple-robot combination systems are correct, and the inverse kinematic correction model with velocity compensation matrix is feasible. Through simulations or experiments, the velocity compensation coefficients of the robots can be measured and the velocity compensation matrix can be created. This modified inverse kinematic model can effectively reduce the errors of robot motion caused by wheel slippage and improve the motion accuracy of the mobile robot system.

## 1. Introduction

In recent years, intelligent and flexible manufacturing has motivated the development of autonomous mobile robots for workpiece and equipment handling and transportation [1,2], and in particular, automated guided vehicle (AGV) technology is widely studied and applied [3,4,5,6,7]. The omni-directional mobile AGV with Mecanum-wheeled robot platform, which has good driving force and easy control performance, can improve the utilization of workshop space and the efficiency of workshop transportation, and is very appropriate for transporting heavy goods in complex industrial environments [8,9]. The AGV that adopts a four-Mecanum-wheeled mobile robot platform with symmetrical structure is the most basic form and most widely used in industry [10,11,12]. In the field of large-scale equipment manufacturing, such as electric multiple unit (EMU) and aircraft, the objects are very heavy and large, and the carrying capacity and size of the four-Mecanum-wheeled AGV cannot meet the transportation requirements. In order to solve the problem, two schemes can be adopted: (1) Using a heavy-duty omnidirectional mobile platform with more Mecanum wheels, such as 8- or 12-wheeled platforms. These robot platforms can also be used in tandem to carry larger loads. For example, German company CLAAS (which has been acquired by MBB Industries AG, and is now Aumann Beelen GmbH) developed an omnidirectional mobile heavy-duty mobile handling robot called MC-Drive, and the MC-Drive TP200 robot has been used to carry aircraft at Airbus manufacturing plants [13,14]. The KUKA omniMove UTV-2 set is a heavy-duty mobile platform with 12 Mecanum wheels that can be used to carry large objects [15]. (2) Using a combination of multiple Mecanum-wheeled robot platforms, which can be considered as a whole with cooperative omnidirectional motion and transport. Usually, mobile platforms used for cooperative transportation are symmetrically arranged. For example, a railcar body can be carried cooperatively by four KUKA omniMove mobile platforms at the Siemens plant in Krefeld, Germany [16]. The four mobile platforms are symmetrically arranged at four corners of the railcar body. Omni-directional mobile platforms with 8, 12, 16, or 32 Mecanum wheels can also be considered as specific combinations of multiple basic mobile platforms.

The mature basic theory of four-Mecanum-wheeled robots is the basis of research on multi-robot systems. Muir [17,18,19] carried out basic research on Mecanum-wheeled robots and developed a kinematic and dynamic model and control on a four-Mecanum-wheeled robot. Campion et al. [20] studied structural properties and classification of kinematic and dynamic models of mobile wheeled robots and derived a motion constraint equation of a Mecanum wheel that can be used in kinematic research of multiple Mecanum-wheeled robots. Robots with four or more Mecanum wheels are overactuated systems with one or more motion constraints (wheel velocities are linearly correlated). Every additional wheel, and every additional robot, adds new motion constraints. So, the motion constraints of a multiple-Mecanum-wheeled robot system can be developed [21], which is also valid for multi-robot systems. The problem of transporting objects with a combined system is also a typical cooperative object transport problem in multi-robot systems, which is a growing research interest in recent years. Tuci et al. [22] provided a comprehensive summary for the scientific literature of cooperative object transport. Alonso-Mora et al. [23] presented a method that exploits deformability during manipulation of soft objects by robot teams including three KUKA YouBot robots. Alonso-Mora et al. [24] presented a constrained optimization method for multi-robot formation control in dynamic environments. Habibi et al. [25,26] presented a scalable distributed path planning algorithm for transporting large objects through unknown environments using a group of homogeneous robots. Lippi et al. [27] studied the modeling and planning problems of a system composed of multiple ground and aerial robots involved in a transportation task. Verginis et al. [28] studied the problem of cooperative transportation of objects rigidly grasped by *N* robotic agents and presented the communication-based decentralized cooperative object transportation using nonlinear model predictive control. Tsai et al. [29] presented a decentralized cooperative transportation control method with obstacle avoidance using fuzzy wavelet neural networks and a consensus algorithm for a group of Mecanum-wheeled omnidirectional robots with uncertainties, in order to move a large payload together. Wang et al. [30] proposed a distributed force and torque controller for a group of robots to collectively transport objects with both translation and rotation control and proved that follower robots can synchronize both their forces and torques with a leader robot that guides the group, and thus contribute positively to the transport. Paniagua-Contro et al. [31] presented an extension of leader–follower behaviors for the case of a combined set of kinematic models of omnidirectional and differential-drive wheeled mobile robots.

Slippage between the wheels and the ground is a disadvantage of Mecanum-wheeled robots, causing them to lose velocity and affecting their positioning accuracy. There are currently some studies on reducing the impact of slippage on the accuracy of robot motion. Chu [32] proposed a method to eliminate position and orientation errors; in this method, multiple ultrasonic distance sensors were used to measure the position and orientation of the mobile robot, and a position compensation algorithm was developed to minimize the position error between the current position and the desired position. Kulkarni et al. [33] proposed a technique to negate wheel slippage by using an additional sensor, a gyroscope, and a nested closed loop control structure to compensate for the increased processing required for slip negation. Tian et al. [34] used a method of back propagation (BP) network for nonlinear motion compensation to reduce the influence of slippage and improve the accuracy of 8 × 8 omnidirectional platform motion. Udomsaksenee et al. [35] proposed a global control method for Mecanum-wheeled vehicles with slip compensation.

The latest research on cooperative transportation mainly focuses on path planning and navigation algorithms in working environments, and control methods in the cooperative transportation process. Research on the Mecanum-wheeled robot platform is mainly focused on single-Mecanum-wheeled robots [8,9,36,37,38,39], while there is less research on the kinematic of a combination system and motion compensation of multiple-Mecanum-wheeled robot platforms. However, studying the kinematic and characteristics of the combined omnidirectional mobile system is the basis for studying the motion control, path planning, and navigation of the combination system. In this research work, we study the combined mobile system of multiple Mecanum-wheeled robots as a whole, not as a cooperative multi-robot system, and the main research interest is the kinematic of this combined system and velocity compensation for the kinematic model, aiming to provide a theoretical basis for motion control of the combined system.

This paper is organized as follows: In Section 2, on the basis of studying the kinematic constraints of a single Mecanum wheel in an omnidirectional mobile system, kinematic models of a four-Mecanum-wheeled robot platform with symmetrical structure and a combined robot system composed of multiple Mecanum-wheeled robots are established, and a motion compensation model is proposed. In Section 3, in order to verify the correctness and feasibility of the kinematic and motion compensation models, the typical motion modes of a virtual prototype of a four-Mecanum-wheeled robot are simulated on RecurDyn. In Section 4, translation motion tests are carried out using a four-Mecanum-wheeled robot physical prototype, and the test results with and without motion compensation are compared. The tests in Section 4 verify the correctness of the motion and motion compensation models and the feasibility of the simulation method in Section 3. In Section 5, the motions of a variety of combined mobile system of a multiple Mecanum-wheeled robot are simulated on RecurDyn, and the kinematic and motion compensation models of a multi-robot system are verified.

## 2. Kinematic Model of Mecanum-Wheeled Mobile Robot

### 2.1. Kinematic Constraint Model of a Single Mecanum Wheel

**Figure 1:**
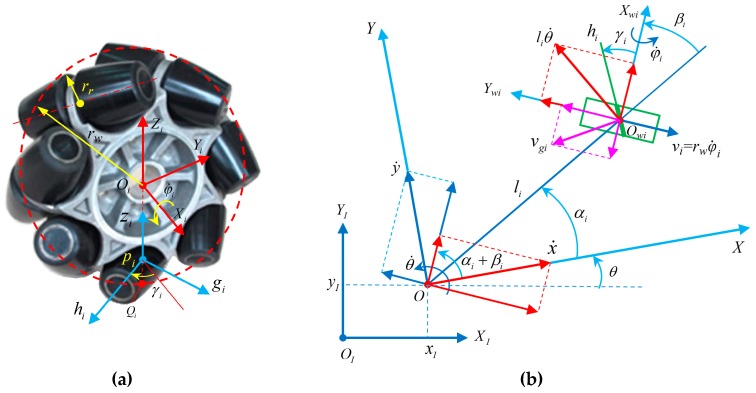
Kinematic constraint diagram of a robotic Mecanum wheel: (**a**) structural principle; (**b**) kinematic constraint.

The kinematic constraints of the *i*-th Mecanum wheel of robot system *O*−*XYZ* consisting of *n* Mecanum wheels are shown in Figure 1 [40,41,42]. 

In Figure 1, the Cartesian coordinate systems of the *i*-th Mecanum wheel and the roller of the wheel are $O_{wi}-X_{wi}Y_{wi}Z_{wi}$ and $P_{i}-g_{i}h_{i}z_{i}$; $r_{w}$ and $r_{r}$ are the radius of the wheel and the roller, respectively; $\left(l_{i},\alpha_{i}\right)$ is used to describe the relative installation orientation of origin O of the body coordinate system and center $O_{i}$ of the wheel; and the angle between the $X_{wi}$ axis and $l_{i}$ is $\beta_{i}$, which is defined as the installation attitude angle of the local coordinate system of the wheel. The velocity of the motion center is $v_{o}$ in the current state, and the angle between $v_{o}$ and the X axis is $\theta_{o}$; $\dot{\theta }$ is the rotation angular velocity of the system when moving in the plane. The angle between the projection of $X_{wi}$ and $h_{i}$ on the plane is the tilt angle $\gamma_{i}$ ($0^{\circ }<\left|\gamma_{i}\right|<90^{\circ }$) of the roller; and $v_{gi}$ is the velocity of the roller touching the ground on the *i*-th Mecanum wheel.

It is assumed that the movement between the roller and the ground is pure rolling, the contact point of the roller with the ground does not slip, and the instantaneous velocity is 0. According to the constraints of rolling and sliding, the following formulas can be obtained [20,43].

(1) $$\left\{ \begin{array}{l} \dot{x}\sin(\alpha_{i}+\beta_{i})-\dot{y}\cos(\alpha_{i}+\beta_{i})-l_{i}\dot{\theta }\cos\beta_{i}=r_{w}{\dot{\varphi }}_{i}-v_{gi}\cos\gamma_{i}\\ \dot{x}\cos(\alpha_{i}+\beta_{i})+\dot{y}\sin(\alpha_{i}+\beta_{i})+l_{i}\dot{\theta }\sin\beta_{i}=-v_{gi}\sin\gamma_{i} \end{array}\right.$$

Because the rollers rotate passively, the velocity of roller $v_{gi}$ is an uncontrollable variable, which is usually not taken into account. By eliminating $v_{gi}$ from Equation (1), we obtain the following [21]:

(2) $$\dot{x}\cos(\alpha_{i}+\beta_{i}+\gamma_{i})+\dot{y}\sin(\alpha_{i}+\beta_{i}+\gamma_{i})+l_{i}\dot{\theta }\sin(\beta_{i}+\gamma_{i})=-r_{w}{\dot{\varphi }}_{i}\sin\gamma_{i}$$

Let $\dot{\zeta }={\left[\begin{array}{ccc} \dot{x} & \dot{y} & \dot{\theta } \end{array}\right]}^{T}$, then the form of the matrix of Equation (2) is as shown in Equation (3), that is, the inverse kinematic equation of any (*i*-th) Mecanum wheel.

(3) $${\dot{\varphi }}_{i}=\frac{-1}{r_{w}\sin\gamma_{i}}\left[\begin{array}{ccc} \cos(\alpha_{i}+\beta_{i}+\gamma_{i}) & \sin(\alpha_{i}+\beta_{i}+\gamma_{i}) & l_{i}\sin(\beta_{i}+\gamma_{i}) \end{array}\right]\dot{\zeta }$$

The motion state of the robot in local coordinate system O−XYZ can be mapped to global coordinate system $O_{I}-X_{I}Y_{I}Z_{I}$, which is expressed as:

(4) $${\dot{\varphi }}_{i}=\frac{-1}{r_{w}\sin\gamma_{i}}\left[\begin{array}{ccc} \cos(\alpha_{i}+\beta_{i}+\gamma_{i}) & \sin(\alpha_{i}+\beta_{i}+\gamma_{i}) & l_{i}\sin(\beta_{i}+\gamma_{i}) \end{array}\right]R(\theta ){\dot{\zeta }}_{I}$$

where ${\dot{\zeta }}_{I}={\left[\begin{array}{ccc} {\dot{x}}_{I} & {\dot{y}}_{I} & {\dot{\theta }}_{I} \end{array}\right]}^{T}$, $R(\theta )=\left[\begin{array}{ccc} \cos\theta & \sin\theta & 0\\ -\sin\theta & \cos\theta & 0\\ 0 & 0 & 1 \end{array}\right]$.

### 2.2. Kinematic Model of Four-Mecanum-Wheeled Mobile Robot with Symmetrical Structure

**Figure 2:**
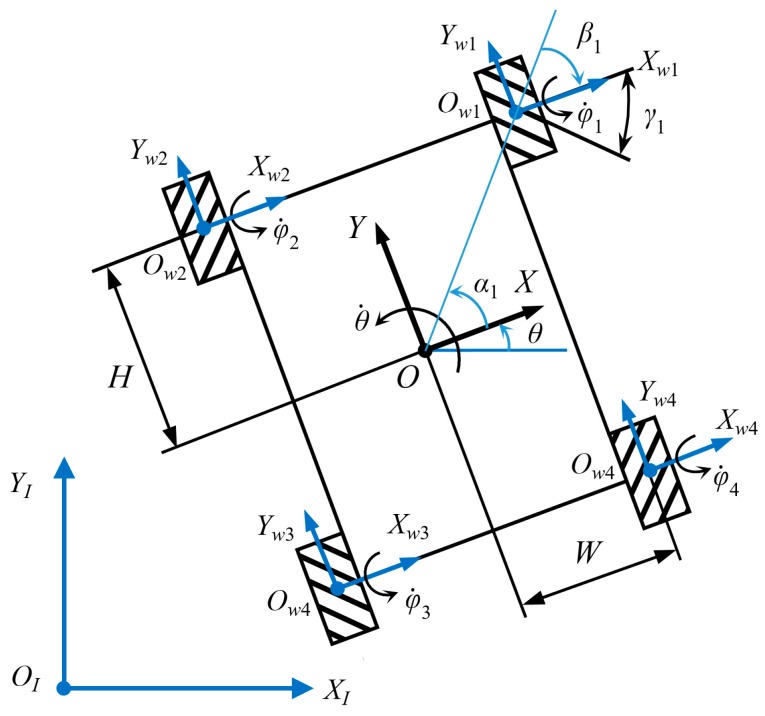
Four-Mecanum-wheeled mobile robot.

In this section, a four-Mecanum-wheeled robot with symmetrical structure is taken as the research object, as shown in Figure 2, and the coordination motion relationship of each wheel is discussed [44,45,46,47]. Choosing the geometrically symmetrical center as system origin *O*, the rectangular coordinate system *XOY* fixed with the mobile platform is established. The coordinate systems of Mecanum wheels $X_{wi}O_{wi}Y_{wi}$ (i=1,2,3,4) are established, taking the wheel centers as the system origins. ${\left[\begin{array}{ccc} \dot{x} & \dot{y} & \dot{\theta } \end{array}\right]}^{T}$ is defined as the generalized velocity of the mobile platform.

According to the geometric characteristics of the robot, for any Mecanum wheel $O_{wi}$, $\alpha_{i}+\beta_{i}=0$. According to Equation (3), the following formula can be obtained.

(5) $${\dot{\varphi }}_{i}=\frac{-1}{r_{w}\sin\gamma_{i}}\left[\begin{array}{ccc} \cos\gamma_{i} & \sin\gamma_{i} & l\sin(\beta_{i}+\gamma_{i}) \end{array}\right]\dot{\zeta }=-\frac{1}{r_{w}}\left[\begin{array}{ccc} \cot\gamma_{i} & 1 & W-H\cot\gamma_{i} \end{array}\right]\left[\begin{array}{l} \dot{x}\\ \dot{y}\\ \dot{\theta } \end{array}\right]$$

where roller angle $\gamma_{1}=\gamma_{3}=-\gamma =-45{}^{\circ}$, $\gamma_{3}=\gamma_{4}=\gamma =45{}^{\circ}$, then the inverse kinematic equation for the four-Mecanum-wheeled robot is [17,18,19,21]:

(6) $$\left[\begin{array}{l} {\dot{\varphi }}_{1}\\ {\dot{\varphi }}_{2}\\ {\dot{\varphi }}_{3}\\ {\dot{\varphi }}_{4} \end{array}\right]=-\frac{1}{r_{w}}\left[\begin{array}{ccc} -\cot\gamma & 1 & W+H\cot\gamma \\ \cot\gamma & 1 & -W-H\cot\gamma \\ -\cot\gamma & 1 & -W-H\cot\gamma \;\\ \cot\gamma & 1 & W+H\cot\gamma \end{array}\right]\left[\begin{array}{l} \dot{x}\\ \dot{y}\\ \dot{\theta } \end{array}\right]=-\frac{1}{r_{w}}\left[\begin{array}{ccc} -1 & 1 & W+H\\ 1 & 1 & -(W+H)\\ -1 & 1 & -(W+H)\;\\ 1 & 1 & W+H \end{array}\right]\left[\begin{array}{l} \dot{x}\\ \dot{y}\\ \dot{\theta } \end{array}\right]$$

The inverse kinematic Jacobian matrix J is expressed as:

(7) $$J=-\frac{1}{r_{w}}\left[\begin{array}{ccc} -1 & 1 & W+H\\ 1 & 1 & -(W+H)\\ -1 & 1 & -(W+H)\;\\ 1 & 1 & W+H \end{array}\right]$$

In the global coordinate system $X_{I}O_{I}Z_{I}$, the inverse kinematic equation of the four-Mecanum-wheeled robot is

(8) $$\left[\begin{array}{l} {\dot{\varphi }}_{1}\\ {\dot{\varphi }}_{2}\\ {\dot{\varphi }}_{3}\\ {\dot{\varphi }}_{4} \end{array}\right]=JR(\theta )\left[\begin{array}{l} {\dot{x}}_{I}\\ {\dot{y}}_{I}\\ \dot{\theta } \end{array}\right]=-\frac{1}{r_{w}}\left[\begin{array}{ccc} -1 & 1 & W+H\\ 1 & 1 & -(W+H)\\ -1 & 1 & -(W+H)\;\\ 1 & 1 & W+H \end{array}\right]\left[\begin{array}{ccc} \cos\theta & \sin\theta & 0\\ -\sin\theta & \cos\theta & 0\\ 0 & 0 & 1 \end{array}\right]\left[\begin{array}{l} {\dot{x}}_{I}\\ {\dot{y}}_{I}\\ \dot{\theta } \end{array}\right]$$

### 2.3. Kinematic Model of Multiple Mecanum-Wheeled Mobile Robot System

**Figure 3:**
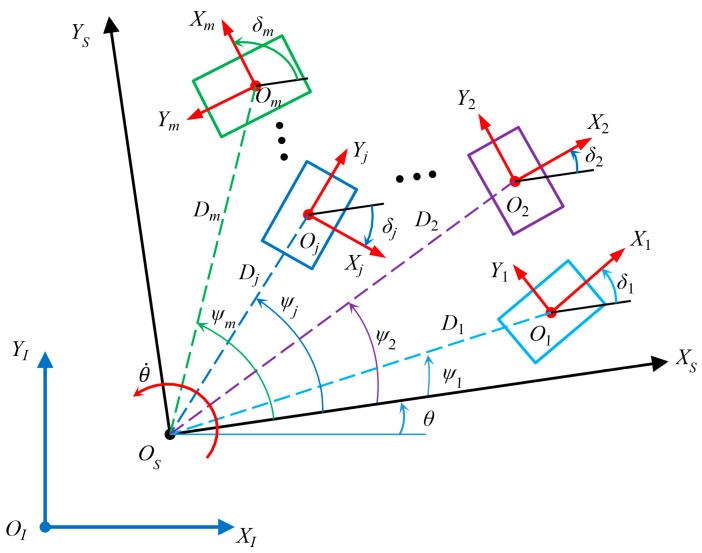
Multiple-robot mobile system.

In Figure 3, in the global coordinate system is $X_{I}O_{I}Y_{I}$, when a multiple Mecanum-wheeled-robot system composed of *m* (m=1,2,⋯j⋯,k) robots co-transports an object, the poses and relative positions of these robots remain unchanged. In order to better describe the motion of the multi-robot system, a common coordinate system $X_{S}O_{S}Y_{S}$ is established at a specified location. $X_{j}O_{j}Y_{j}$ is the local coordinate system of the *j*-the robot of this system, which consists of $n_{j}$ ($n_{j}=1,2,\cdots i\cdots ,k$) Mecanum wheels. The relative position of coordinate systems $X_{S}O_{S}Y_{S}$ and $X_{j}O_{j}Y_{j}$ of each robot is determined by the geometric parameters of the multi-robot system. Let ${\dot{\Phi }}_{jn_{j}}$, $J_{j}$, and ${\dot{\zeta }}_{j}$ be the wheel rotation matrix, Jacobian matrix, and motion state in the local coordinate system of the *j*-th robot, respectively. The inverse kinematic equation of the *j*-th robot is shown in Equation (9):

(9) $${\dot{\Phi }}_{jn_{j}}=J_{j}{\dot{\zeta }}_{j}$$

After transforming the local coordinate system $X_{j}O_{j}Y_{j}$ of the *j*-th robot to the designated coordinate system $X_{S}O_{S}Y_{S}$, set ${\dot{\zeta }}_{S}={\left[\begin{array}{ccc} {\dot{x}}_{S} & {\dot{y}}_{S} & \dot{\theta } \end{array}\right]}^{T}$, and the inverse kinematic equation of the robot is as follows:

(10) $${\dot{\Phi }}_{jn_{j}}=J_{j}K_{j}{\dot{\zeta }}_{S}$$

where

(11) $$\begin{array}{ll} K_{j} & =R(\delta_{j})T(D_{j},\psi_{j})=\left[\begin{array}{ccc} \cos\delta_{j} & \sin\delta_{j} & 0\\ -\sin\delta_{j} & \cos\delta_{j} & 0\\ 0 & 0 & 1 \end{array}\right]\left[\begin{array}{ccc} 1 & 0 & -D_{j}\sin\psi_{j}\\ 0 & 1 & D_{j}\cos\psi_{j}\\ 0 & 0 & 1 \end{array}\right]\\ & =\left[\begin{array}{ccc} \cos\delta_{j} & \sin\delta_{j} & D_{j}\sin(\delta_{j}-\psi_{j})\\ -\sin\delta_{j} & \cos\delta_{j} & D_{j}\cos(\delta_{j}-\psi_{j})\\ 0 & 0 & 1 \end{array}\right] \end{array}$$

The inverse kinematic equation of the *j*-th robot in global coordinate system $X_{I}O_{I}Y_{I}$ is

(12) $${\dot{\Phi }}_{jn_{j}}=J_{j}K_{j}R(\theta ){\dot{\zeta }}_{I}$$

In global coordinate system $X_{I}O_{I}Y_{I}$, the inverse kinematic equation of the multi-robot mobile system composed of *m* (m=1,2,⋯j⋯,k) robots is shown as follows:

(13) $$\left[\begin{array}{c} {\dot{\Phi }}_{1n_{1}}\\ {\dot{\Phi }}_{2n_{2}}\\ \vdots \\ {\dot{\Phi }}_{jn_{j}}\\ \vdots \\ {\dot{\Phi }}_{mn_{m}} \end{array}\right]=\left[\begin{array}{c} J_{1}K_{1}\\ \begin{array}{c} J_{2}K_{2}\\ \vdots \\ J_{j}K_{j} \end{array}\\ \vdots \\ J_{m}K_{m} \end{array}\right]R(\theta )\left[\begin{array}{c} {\dot{x}}_{I}\\ {\dot{y}}_{I}\\ \dot{\theta } \end{array}\right]$$

If the multiple-robot system only performs translational motion, its inverse kinematic equation is as follows

(14) $$\left[\begin{array}{c} {\dot{\Phi }}_{1n_{1}}\\ {\dot{\Phi }}_{2n_{2}}\\ \vdots \\ {\dot{\Phi }}_{jn_{j}}\\ \vdots \\ {\dot{\Phi }}_{mn_{m}} \end{array}\right]=\left[\begin{array}{c} J_{1}K_{1}\\ \begin{array}{c} J_{2}K_{2}\\ \vdots \\ J_{j}K_{j} \end{array}\\ \vdots \\ J_{m}K_{m} \end{array}\right]\left[\begin{array}{c} {\dot{x}}_{I}\\ {\dot{y}}_{I}\\ 0 \end{array}\right]$$

**Figure 4:**
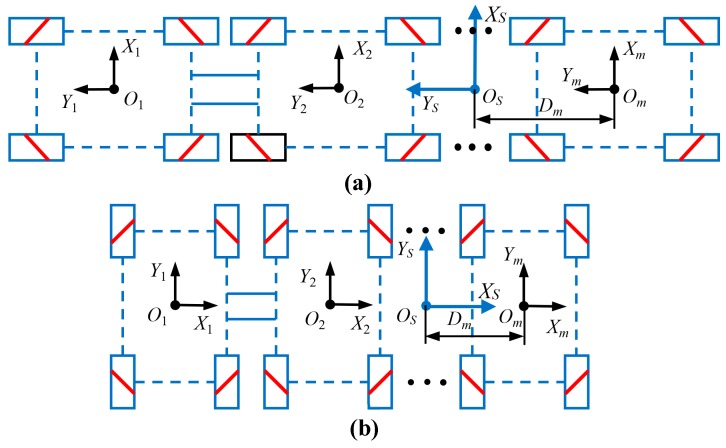
Combination configurations of multiple four-Mecanum-wheeled mobile platform with symmetrical structure: (**a**) tandem connected configuration; (**b**) side-by-side connected configuration.

The robot configuration composed of *m* same four-Mecanum-wheeled robots connected end-to-end and side-by-side is a common multi-robot system, as shown in Figure 4. The coordinate system $X_{S}O_{S}Y_{S}$ of the robot system is usually established on the structurally symmetric center line. ${\dot{\Phi }}_{j4}$, J, and $K_{j}$ are the wheel velocity matrix, Jacobian matrix, and motion state transformation matrix, respectively, relative to the specified reference coordinate system $X_{S}O_{S}Y_{S}$ of the *j*-th four-Mecanum-wheeled robot. The inverse kinematic equation of the multi-robot configuration is as follows:

(15) $$\left[\begin{array}{c} {\dot{\Phi }}_{14}\\ {\dot{\Phi }}_{24}\\ \vdots \\ {\dot{\Phi }}_{j4}\\ \vdots \\ {\dot{\Phi }}_{m4} \end{array}\right]=\left[\begin{array}{c} JK_{1}\\ JK_{2}\\ \vdots \\ JK_{j}\\ \vdots \\ JK_{m} \end{array}\right]\left[\begin{array}{c} {\dot{x}}_{S}\\ {\dot{y}}_{S}\\ \dot{\theta } \end{array}\right]=\left[\begin{array}{c} JT(D_{1},\psi_{1})\\ JT(D_{2},\psi_{2})\\ \vdots \\ JT(D_{j},\psi_{j})\\ \vdots \\ JT(D_{m},\psi_{m}) \end{array}\right]R(\theta )\left[\begin{array}{c} {\dot{x}}_{I}\\ {\dot{y}}_{I}\\ \dot{\theta } \end{array}\right]$$

where ${\dot{\Phi }}_{j4}={\left[\begin{array}{cccc} {\dot{\varphi }}_{j1} & {\dot{\varphi }}_{j2} & {\dot{\varphi }}_{j3} & {\dot{\varphi }}_{j4} \end{array}\right]}^{T}$, $\psi_{j}=\pm 90^{\circ }$.

### 2.4. Velocity Compensation of the Robot System

During the movement of the robot, due to manufacturing errors, wheel slippage, and other factors, there are errors between the actual velocity and the desired velocity, and the relative errors of longitudinal, lateral, and rotational angular speed are also different. Normally, the actual velocity of the robot is less than the set desired velocity. Different robots have different wheel slip rates and velocity errors due to different structural dimensions, manufacturing precision, number of wheels, deformation rate of wheel rollers, and friction coefficient between wheel and ground. For example, the precision of the roller angle of the Mecanum wheel has a great influence on the motion accuracy and velocity of the robot. In the following, taking a four-Mecanum-wheeled robot as an example, the influence of the precision of the roller angle on velocity is analyzed.

In order to analyze the influence of roller angle γ on the velocity of the robot, the partial derivative of Equation (6) with respect to γ is calculated.

(16) $$\left[\begin{array}{l} \frac{\partial {\dot{\varphi }}_{1}}{\partial \gamma }\\ \frac{\partial {\dot{\varphi }}_{2}}{\partial \gamma }\\ \frac{\partial {\dot{\varphi }}_{3}}{\partial \gamma }\\ \frac{\partial {\dot{\varphi }}_{4}}{\partial \gamma } \end{array}\right]=-\frac{1}{r_{w}}\left[\begin{array}{ccc} \csc^{2}\gamma & 0 & -H\csc^{2}\gamma \\ -\csc^{2}\gamma & 0 & H\csc^{2}\gamma \\ \csc^{2}\gamma & 0 & H\csc^{2}\gamma \;\\ -\csc^{2}\gamma & 0 & -H\csc^{2}\gamma \end{array}\right]\left[\begin{array}{l} \dot{x}\\ \dot{y}\\ \dot{\theta } \end{array}\right]$$

If there is an error in roller angle Δγ and $\gamma =45^{\circ }$, the wheel velocity will be compensated according to Equation (17):

(17) $$\left[\begin{array}{l} \Delta {\dot{\varphi }}_{1}\\ \Delta {\dot{\varphi }}_{2}\\ \Delta {\dot{\varphi }}_{3}\\ \Delta {\dot{\varphi }}_{4} \end{array}\right]=-\frac{\Delta \gamma }{r_{w}}\left[\begin{array}{ccc} \csc^{2}\gamma & 0 & -H\csc^{2}\gamma \\ -\csc^{2}\gamma & 0 & H\csc^{2}\gamma \\ \csc^{2}\gamma & 0 & H\csc^{2}\gamma \;\\ -\csc^{2}\gamma & 0 & -H\csc^{2}\gamma \end{array}\right]\left[\begin{array}{l} \dot{x}\\ \dot{y}\\ \dot{\theta } \end{array}\right]=-\frac{\Delta \gamma }{r_{w}}\left[\begin{array}{ccc} 2 & 0 & -2H\\ -2 & 0 & 2H\\ 2 & 0 & 2H\;\\ -2 & 0 & -2H \end{array}\right]\left[\begin{array}{l} \dot{x}\\ \dot{y}\\ \dot{\theta } \end{array}\right]$$

According to Equation (17), the longitudinal motion velocity of the robot is not related to the roller angle, so it is unnecessary to compensate the wheel velocity, the lateral velocity and rotation velocity need to be compensated; and the rotation velocity compensation rate is related to the structural parameters of the robot. If the angle error of each roller of each Mecanum wheel is different, the situation will be more complicated. Because the velocity error is the result of many factors, the contribution of each factor is difficult to measure accurately. For a specific robot, its geometric size, manufacturing error, wheel deformation, and friction coefficient with the designated ground have been determined. The influence of these factors on the speed error can be determined. The velocity error may have a certain functional relationship with the motion variables, such as velocity of the robot. At different velocities, the relative errors of the robot’s longitudinal, transverse, and rotational velocity can be obtained by experiments, so that the velocity compensation coefficients with different velocities can be obtained. For the multi-robot system in Figure 3, the velocity compensation equation matrix of the *j*-th robot is:

(18) $$C_{j}=\left[\begin{array}{ccc} f_{j} & 0 & 0\\ 0 & g_{j} & 0\\ 0 & 0 & u_{j} \end{array}\right]$$

Then, the inverse kinematic equation of the *j*-th robot with velocity compensation is

(19) $${{\dot{\Phi }}^{\prime }}_{jn_{j}}=J_{j}C_{j}{\dot{\zeta }}_{j}=J_{j}C_{j}K_{j}{\dot{\zeta }}_{S}=J_{j}C_{j}K_{j}R(\theta ){\dot{\zeta }}_{I}$$

The inverse kinematic equation of this multi-robot system composed of m robots with velocity compensation is

(20) $$\left[\begin{array}{c} {{\dot{\Phi }}^{\prime }}_{1n_{1}}\\ {{\dot{\Phi }}^{\prime }}_{2n_{2}}\\ \vdots \\ {{\dot{\Phi }}^{\prime }}_{jn_{j}}\\ \vdots \\ {{\dot{\Phi }}^{\prime }}_{mn_{m}} \end{array}\right]=\left[\begin{array}{c} J_{1}C_{1}K_{1}\\ \begin{array}{c} J_{2}C_{2}K_{2}\\ \vdots \\ J_{j}C_{j}K_{j} \end{array}\\ \vdots \\ J_{m}C_{m}K_{m} \end{array}\right]R(\theta )\left[\begin{array}{c} {\dot{x}}_{I}\\ {\dot{y}}_{I}\\ \dot{\theta } \end{array}\right]$$

If the multi-robot system only performs translational motion, the inverse kinematic equation with velocity compensation is

(21) $$\left[\begin{array}{c} {{\dot{\Phi }}^{\prime }}_{1n_{1}}\\ {{\dot{\Phi }}^{\prime }}_{2n_{2}}\\ \vdots \\ {{\dot{\Phi }}^{\prime }}_{jn_{j}}\\ \vdots \\ {{\dot{\Phi }}^{\prime }}_{mn_{m}} \end{array}\right]=\left[\begin{array}{c} J_{1}C_{1}K_{1}\\ \begin{array}{c} J_{2}C_{2}K_{2}\\ \vdots \\ J_{j}C_{j}K_{j} \end{array}\\ \vdots \\ J_{m}C_{m}K_{m} \end{array}\right]\left[\begin{array}{c} {\dot{x}}_{I}\\ {\dot{y}}_{I}\\ 0 \end{array}\right]$$

For the four-Mecanum-wheeled robot in Figure 2, according to Equations (8) and (19), the inverse kinematic equation with velocity compensation is

(22) $$\left[\begin{array}{l} {{\dot{\varphi }}^{\prime }}_{1}\\ {{\dot{\varphi }}^{\prime }}_{2}\\ {{\dot{\varphi }}^{\prime }}_{3}\\ {{\dot{\varphi }}^{\prime }}_{4} \end{array}\right]=JCR(\theta )\left[\begin{array}{l} {\dot{x}}_{I}\\ {\dot{y}}_{I}\\ \dot{\theta } \end{array}\right]$$

## 3. Motion Simulation of Four-Mecanum-Wheeled Robot Virtual Prototype and Motion Test of Physical Robot

In order to further verify the correctness of the above theoretical research, the Mecanum-wheeled robot was simulated and analyzed by virtual prototyping technology. In this paper, the virtual prototype of the Mecanum-wheel mobile platform was modelled in SolidWorks, and the simulation was carried out on RecurDyn.

### 3.1. Creating a Four-Mecanum-Wheeled Robot Virtual Prototype for Simulation

#### 3.1.1. Importing Robot Virtual Prototype Model and Creating Joints and Motions

Before importing into RecurDyn, the robot model should be simplified. The simplified assembly model built in SolidWorks should be imported into RecurDyn in Parasolid format. In the process of modeling, a single Mecanum wheel is defined as a subsystem in RecurDyn, which not only facilitates the hierarchical management of the model, but also enables the subsystem to be established, modified, imported, and exported independently. That can improve the reuse rate of the model, reduce the chance of model construction errors, and improve the efficiency. 

**Figure 5:**
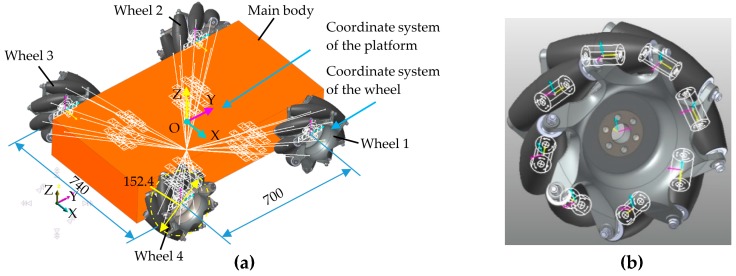
Simulation model of four-Mecanum-wheeled robot platform: (**a**) 3D model; (**b**) 8 revolute joints between rollers and hub in single Mecanum wheel subsystem.

According to the actual movement of the robot platform, the constraint relationship between components is created based on determining the assembly and motion relationships of components. In the robot virtual prototype simulation model, 36 revolute joints are created, as shown in Figure 5a. There are four revolute joints between the hub of the Mecanum wheel and the main body, and eight revolute joints between the rollers and the hub in a single Mecanum wheel subsystem, as shown in Figure 5b. The following are the basic dimensions of the robot model: γ = 45°, W = 370 mm, H = 350 mm, and R = 152.4 mm. The weight of the robot is 200 kg. By setting the angular velocity motion functions on the revolute joints between the four Mecanum wheels and the main body, the robot platform can move in simulation at desired speed. In order to prevent system anomalies caused by sudden changes in speed during the simulation, the drive function is usually applied to the revolute joints using the system’s predefined STEP function. The STEP function is a third-order polynomial function built into the software and is used to define a relatively smooth load curve, which is suitable for smoothly loading drivers for mobile robot platforms.

#### 3.1.2. Creating Contacts and Setting Parameters

Only by adding contacts between the rollers and the ground and setting reasonable contact parameters can the virtual prototype be simulated in accordance with the predetermined scheme. The RecurDyn contact toolkit provides rich contact models, from which Solid Contact was selected. Usually, the outer layer of the roller is vulcanized with a layer of rubber or polyurethane material. The higher the surface hardness of the roller, the stronger the bearing capacity and the higher the elastic modulus. The surface hardness of rollers with Mecanum wheels for small mobile platforms is usually 75 A Shaw hardness, and 90 A for the rollers of heavy-duty AGV. Moreover, we assume that the workplace has C30 grade concrete ground. The estimated values of the attribute parameters of the roller and the ground are shown in Table 1.

Based on Hertz contact theory, the stiffness coefficient between two objects can be expressed as:

(23) $$k=\frac{4}{3}\rho^{\frac{1}{2}}E^{*}$$

where ρ is the comprehensive radius of curvature, $\frac{1}{\rho }=\frac{1}{\rho_{1}}+\frac{1}{\rho_{2}}$; and $\rho_{1}$ and $\rho_{2}$ are the radii of curvature of the two objects at the contact; and $E^{*}$ is the comprehensive elastic modulus; $\frac{1}{E^{*}}=\frac{1-\upsilon_{1}^{2}}{E_{1}}+\frac{1-\upsilon_{2}^{2}}{E_{2}}$; $E_{1}$, $E_{2}$ and $\upsilon_{1}$, $\upsilon_{2}$ are the elastic modulus and Poisson’s ratio of the two materials, respectively.

The material property parameters of the ground and the rollers in Table 1, the radius ($\rho_{1}=10^{10}\;\mathrm{mm}$) of curvature of the horizontal ground, and the radius ($\rho_{2}=33.5\;\mathrm{mm}$) of curvature of the roller are substituted into Equation (23), and the stiffness coefficient between the two objects can be calculated. According to the theoretical calculation and feedback of simulation results, the contact parameters are set as follows: spring coefficient is 1200 N/mm, damping coefficient is 4, dynamic friction coefficient is 0.35, stiffness exponent is 2, and the rest are kept as default. On each roller of the Mecanum wheel, solid contact with the ground is set. Each Mecanum wheel is provided with 8 solid contacts on the ground, so 32 solid contacts are established for the mobile platform. The virtual prototype simulation model established through the above steps is shown in Figure 5a.

### 3.2. Motion Simulation and Analysis of a Single Four-Mecanum-Wheeled Robot

**Figure 6:**
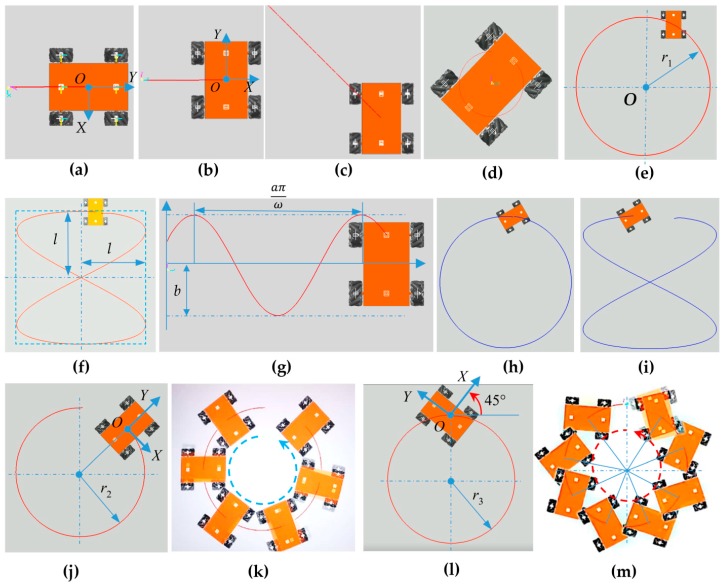
Motion simulation of robot: (**a**) longitudinal motion; (**b**) lateral motion; (**c**) oblique motion at 45°; (**d**) turning on the spot; (**e**) circular motion, where radius *r*_1_ of the circle is 2500 mm; (**f**) translation motion around 8-figure curve; (**g**) translational motion along a simple harmonic motion curve; (**h**) circular translation motion of robot in oblique state; (**i**) translation motion of robot around 8-figure curve in oblique state; (**j**,**k**) centripetal circular motion, where radius *r*_2_ of the circle is 1350 mm; (**l**,**m**) centripetal motion of 45° angle, where radius *r*_3_ of the circle is 1350 mm.

The motion simulations of the four-Mecanum-wheeled robot platform were carried out using 11 modes to analyze the motion characteristics. In order to better show the motion state of the robot platform during the simulation process, the trajectory tool in the software was used to display the motion track of the robot center or the specified marker point. The motion situation and trajectories of the robot in the simulation using 11 motion modes are shown in Figure 6. The simulation trajectories of the center of the robot platform in longitudinal, lateral, and oblique motion at 45° are shown in Figure 6a–c, respectively. Figure 6d–g show screenshots of the motion simulation including turning on the spot, circular motion, moving around an 8-figure curve, and sinusoidal curve motion. Figure 6h,i show the robot moving along a circle and an 8-figure curve in the oblique state. Figure 6j,k show centripetal circular motion. Figure 6l,m show centripetal motion of a 45° angle. The centripetal circular motion is a combination of translation and rotation. The curve equations of the robot moving in circular, 8-shaped, and harmonic curves are shown in Table 2.

#### 3.2.1. Analysis of Simulations of Four Simple Motions

(1) Longitudinal and Lateral Motion Simulation

**Figure 7:**
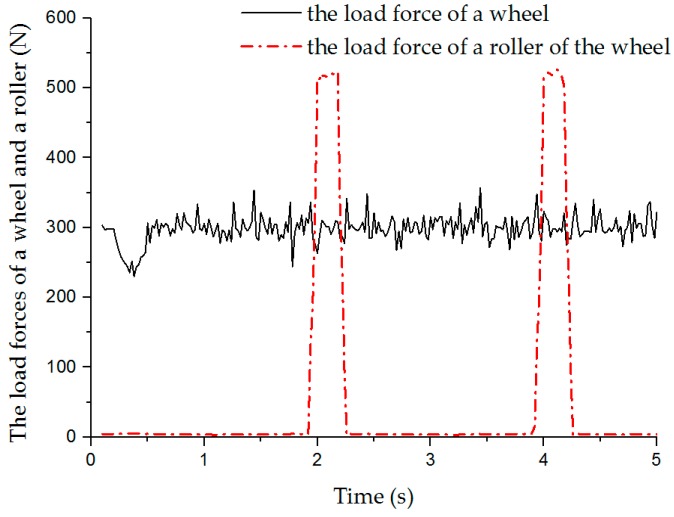
Wheel load and roller load in longitudinal motion simulation.

**Figure 8:**
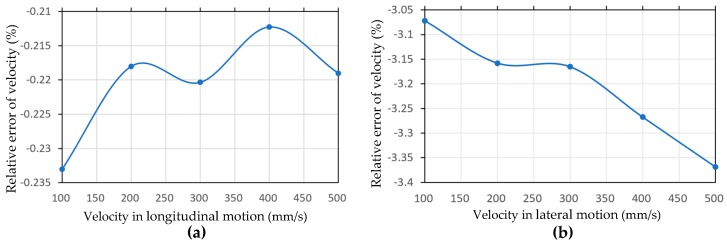
Relative error of velocity in (**a**) longitudinal and (**b**) lateral translation motion simulation.

Figure 7 shows the load curves of a wheel and a roller on the wheel during longitudinal movement of the mobile platform. By observing these two load curves, it can be seen that both the wheel and the roller bear periodic loads. The periodic load on the roller is derived from the roller being in contact with the ground every revolution of the wheel, and the contact duration is short and the load changes drastically. When the robot platform is in a stable motion state, the periodicity of the wheel load is mainly affected by the change of the state of contact between the roller and the ground on the body. In addition, the load on the roller is significantly greater than the load on the wheel, since the load on the entire robot is all applied to the roller in contact with the ground. The longitudinal and lateral motions of the robot are simulated with a series of different velocities, and the average velocities of the robot in stable state are obtained, as shown in Table 3. These simulation velocities are less than the corresponding set velocities. From the simulation data, the relative error of longitudinal velocity is very small; the average relative error is about −0.22%, so its correlation with velocity is not obvious. Five sets of simulation results show that the velocity loss of lateral translation is significantly greater than that of longitudinal translation when the robot runs at the same set velocity. Moreover, there is a negative correlation between the error of the robot and the set velocity in the lateral translation motion. The relative error curves of the velocities of the robot in the two motion modes are shown in Figure 8.

**Figure 9:**
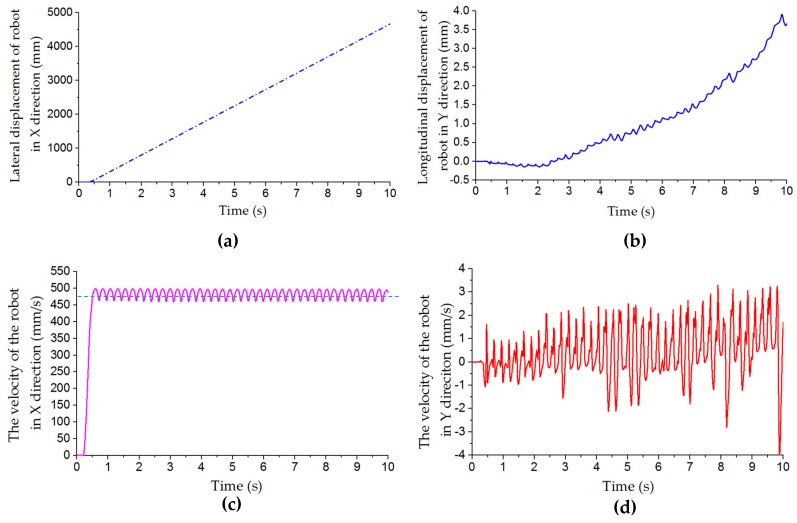
Displacement and velocity curves of robot in x- and y-directions during lateral motion simulation: (**a**) lateral displacement curve in x-direction; (**b**) longitudinal displacement curve in y-direction; (**c**) velocity curve in x-direction; (**d**) velocity curve in y-direction.

Figure 9a shows the displacement curve of the robot in the x-direction in lateral motion. Figure 9c shows the x- direction velocity curve of the robot platform when it moves laterally at a set velocity of 500 mm/s. In the figure, the velocity value rises gradually from zero, which reflects the process of accelerating the robot platform from static state to stable moving state, and then the value fluctuates steadily in a range. In the simulation of lateral movement at a set speed of 500 mm/s, the maximum lateral velocity is 497.5 mm/s, the minimum velocity is 460.4 mm/s, and the average velocity is 483.2 mm/s. Figure 9b shows the longitudinal displacement and velocity curves in the y-direction during lateral motion. When the robot moves laterally in the x-direction, it fluctuates slightly in the y-direction and deviates from the x-axis direction. The velocity of the robot along the y-axis fluctuates around the zero line, as shown in Figure 9d. In 10 seconds of lateral movement, that is, when the robot moves about 5000 mm in the lateral direction, it deviates from the x-axis direction by about 4 mm. So, the longitudinal offset has little effect on the lateral motion of the robot platform.

(2) Simulation of Oblique Motion at 45°

When the robot moves in a straight line with an oblique direction of 45°, only two Mecanum wheels on one diagonal line rotate; the wheels on the other diagonal line do not rotate, but the bottom rollers on the wheels that do not turn passively rotate. Table 4 shows the simulation velocities and relative errors of the 45° oblique translation of the robot. According to the simulation data, there is loss of velocity in motion, and the velocity loss in the x- and y-directions is almost equal. The relative error of velocity in the x-direction is smaller than that of the lateral translation set at the same velocity.

**Figure 10:**
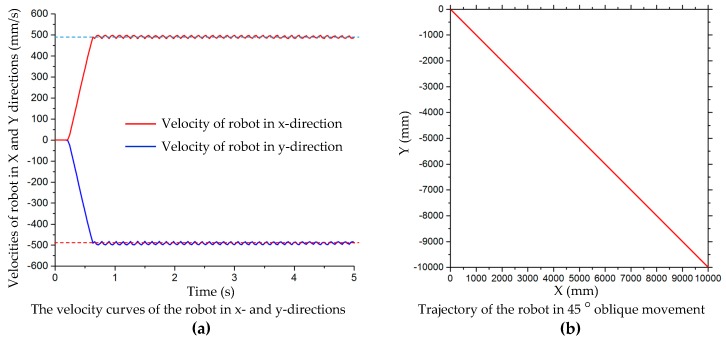
(**a**) Velocity curves in x- and y-directions, and (**b**) trajectory of robot platform in simulation of oblique motion at 45°.

Figure 10a shows that the velocity of the x- and y-axis increases rapidly from 0 to a predetermined value within 0.6 s when the mobile platform moves along the oblique direction of 45°. Then the velocities in the two directions are basically the same, and fluctuate within a certain range. In the simulation, the velocity in the x- and y-axis directions is set at 500 mm/s, and the average test velocities are 490.164 mm/s and 490.061 mm/s, respectively. The curves of the two velocities are symmetrical relative to the zero line. The combination of these two directions enables the robot platform to move along the 45° direction. The trajectory curve of the robot in the oblique 45° motion is shown in Figure 10b.

(3) Turning on the Spot

**Figure 11:**
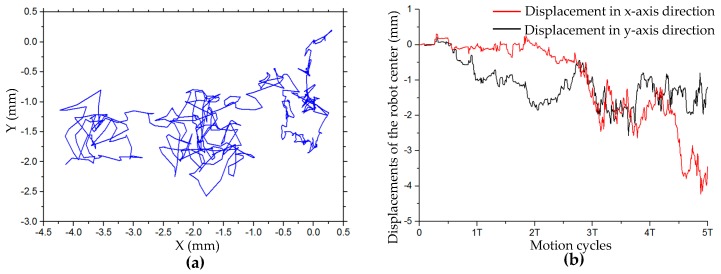
(**a**) Trajectory and (**b**) displacement of robot center in x- and y-axis directions in simulation of turning on the spot in five cycles.

Table 5 shows the relative errors of the angular velocity of the robot in the turning on the spot motion simulation at different angular velocities, which are positively correlated with increased velocity. The trajectory of the robot center in this simulation in five cycles is shown in Figure 11a. The trajectory has great randomness. In the first cycle, the displacement of the robot center in both x- and y-axis directions is within 1 mm. However, with the increased number of rotation cycles, the displacement tends to increase. After five cycles of rotation, the maximum displacement of the robot has exceeded 4 mm. The displacement curves of the robot center in the x- and y-axis directions in five cycles are shown in Figure 11b. It can be seen that the offset of the center coordinate of the robot increases with the increased number of rotation cycles.

According to the errors between simulation results and theoretical values of the velocities shown in Table 3, Table 4 and Table 5, the simulation velocities of the robot are less than the theoretical velocities. In these motion modes, the relative errors of simulation velocity vary slightly with different velocities but have a positive correlation on the whole, especially in lateral translation, oblique 45° translation, and in situ rotation. Comparing these three kinds of motion simulation, the velocity error of lateral motion is the largest, and that of longitudinal motion is the smallest. The relative error of velocity of 45° oblique motion is smaller than that of lateral motion and more than that of longitudinal motion. The relative error of angular velocity of in situ rotation is equivalent to that of 45° oblique translation motion.

Possible reasons for the errors between the simulation and theoretical set velocity are as follows: First, the wheel skidding phenomenon occurs in the process of rotation, which is common in wheeled mobile robots; slipping is most serious in the process of lateral movement. Second, the rim of the multiple rollers of the Mecanum wheel is theoretically a circle; however, in the simulation movement, deformation of the rubber layer at different positions of the rollers under load is different, and the effective radius of the wheel changes with rotation, thereby causing periodic changes even if the angular velocity is the same. Third, the roller cuts into the ground under load, which causes the radius of the wheel to be less than the theoretical radius, which is similar to the compression of the elastic roller in actual motion.

It can be seen that there are errors between the robot’s velocity in motion simulation and the set velocity, especially in lateral translation motion. In order to ensure precise motion of the robot along the desired trajectory, it is necessary to compensate its velocity.

#### 3.2.2. Velocity Compensation in Motion Simulation of Complex Trajectories

**Figure 12:**
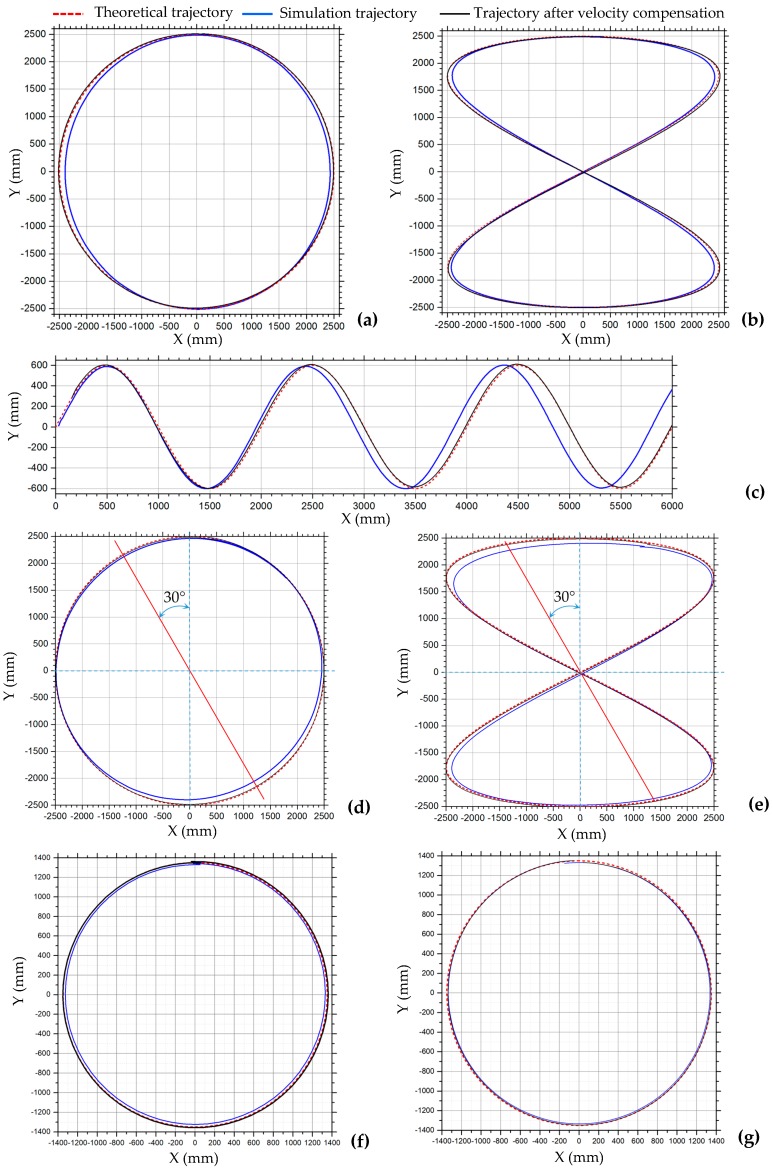
Trajectories of robot in motion simulation shown in Figure 6: (**a**) circular trajectory in Figure 6e; (**b**) 8-shaped trajectory in Figure 6f; (**c**) simple harmonic motion trajectory in Figure 6g; (**d**) circular trajectory in Figure 6h; (**e**) 8-shaped trajectory in Figure 6i; (**f**) centripetal circular motion in Figure 6j; (**g**) centripetal motion of 45° angle in Figure 6l.

According to the simulation results of simple motion in the previous section, it can be seen that there are velocity losses in longitudinal translation, lateral translation, and rotational motion. When the robot translates along a complex trajectory, such as circular, 8-shaped, and simple harmonic motion curves, its motion can be decomposed into motion in both x- and y-directions. When the robot performs simulated motion along complex trajectories, if a theoretical velocity value is assigned to each Mecanum wheel, the robot will not be able to move to the expected position, so there is an error between the simulated and theoretical trajectory. The trajectories of the robot in the motion simulation along circular, 8-shaped, and harmonic curves are plotted with blue lines in Figure 12. These simulation curves are basically consistent with the theoretical curves, plotted with dashed red lines in Figure 12, but because of the velocity error of the robot, there are deviations between the simulation and theoretical curves. The circular and 8-shaped simulation curves are closed, so the curves deviate inward. The displacement errors of circular curves in Figure 12a in the x- and y-axis directions are 3.48% and 0.14%, respectively. The displacement errors of the 8-shaped trajectory in Figure 12b in the x- and y-axis are 2.92% and 0.28%, respectively. Relative errors of displacement of x- and y-axis directions in motion simulation along the circular and 8-shaped curves are shown in Table 6. With the increased motion period, the deviation of the simple harmonic trajectory in the x-direction increases, as shown in Figure 12c. If the robot keeps a certain tilt angle during the translational motion, there is a large displacement error in the trajectory in the lateral direction. Figure 12d,e show the trajectories of the robot in motion simulation shown in Figure 6h,i, maintaining a 60° angle during translating motion. According to Figure 12d,e, the deviation between the robot’s trajectory and the theoretical curve in the oblique direction is the largest. The oblique direction is the x-direction in the local coordinate system of the robot.

Compensating the robot’s velocity can reduce the trajectory error and make the trajectory closer to the theoretical trajectory. In the translational motion of the robot, only the velocity in the x- and y-axis directions needs to be compensated. According to the simulation data in Table 3, as the velocity changes, the relative error of the robot’s velocity changes, but the amplitude is small. When the robot makes a curve motion, the velocities of the robot and the wheels change with the moment of change at all times. So a fixed compensation coefficient can be set for the virtual prototype model of the robot. In this simulation test, the average values of the velocity compensation coefficients of the longitudinal and lateral translation motions in Table 3 are used as the compensation coefficients in the x- and y-axis directions. The motion shown in Figure 6e–i is translation motion; according to Table 3, the velocity compensation matrix is set to *C*_1_. The centripetal circular motion shown in Figure 6j–m is a combination of translation and rotation; therefore, it is also necessary to compensate for its rotational motion. According to Table 5, the compensation coefficient of rotational angular velocity is set to 1.0195, and the compensation coefficient of lateral velocity is set to 1.04 due to its large simulated lateral velocity. Then, the compensation matrix of velocity is set to *C*_2_.

$$C_{1}=\left[\begin{array}{ccc} f & 0 & 0\\ 0 & g & 0\\ 0 & 0 & u \end{array}\right]=\left[\begin{array}{ccc} 1.033 & 0 & 0\\ 0 & 1.002 & 0\\ 0 & 0 & 0 \end{array}\right],\;C_{2}=\left[\begin{array}{ccc} 1.04 & 0 & 0\\ 0 & 1.002 & 0\\ 0 & 0 & 1.0195 \end{array}\right]$$

The trajectories of the robot in the motion simulation along the circular, 8-shaped, and simple harmonic motion curves after the velocity compensation are plotted with solid black lines in Figure 12. It can be seen from Figure 12a–e that the trajectories after velocity compensation are very consistent with the theoretical curves. The trajectories of the centripetal circular motions in Figure 6j–m are shown in Figure 12f,g, and the simulation trajectories after velocity compensation are better than those without velocity compensation. The simulation results show that this velocity compensation method is effective.

## 4. Motion Test of a Four-Mecanum-Wheeled Robot Physical Prototype

### 4.1. Motion Test System of Mecanum-Wheeled Robot Using Optitrack Optical Motion Capture System

**Figure 13:**
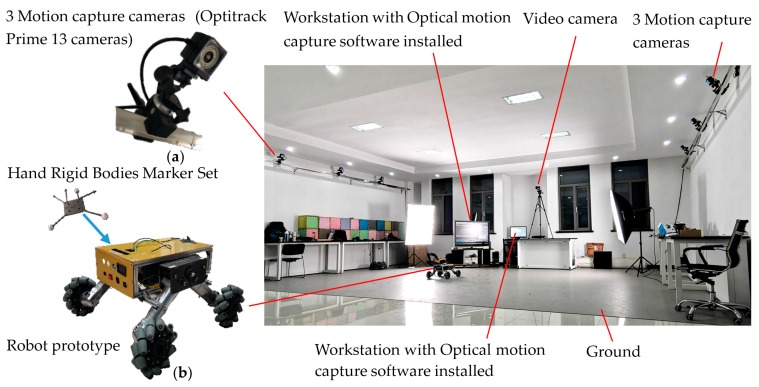
Test system of the Mecanum-wheeled robot using the Optitrack optical motion capture system: (**a**) the Optitrack Prime 13 cameras; (**b**) four-Mecanum-wheeled robot prototype.

In Section 3, RecurDyn software was used to simulate the robot’s motion, and the motion simulations verified the correctness of the kinematic model of the four-Mecanum-wheeled robot and the feasibility of velocity compensation. However, they were carried out to simulate an ideal robot on ideal ground, which is quite different from real situations. The movement of a robot in a real environment is affected by many factors, including machining error of the Mecanum wheels, angle error of rollers, installation error of the four wheels, flatness of the ground, and control performance of the motor. In order to verify the correctness of the motion and motion compensation models, motion tests of the robot physical prototype were carried out. In order to measure the coordinates and trajectories of the robot during movement, the motion test system was constructed using the OptiTrack optical motion capture system, as shown in Figure 13 [48]. Three OptiTrack Prime 13 cameras (high-speed motion capture cameras), shown in Figure 13a, were arranged on each side of the test area. The cameras were connected to the data and power supply by using a Gigabit Ethernet GigE/PoE interface. All cameras were connected to a Gigabit network hub with Ethernet cables. An installed workstation with Motive optical motion capture was connect to the hub with a cable. The Motive software was used for recording, presentation, playback, and remote data services of the position data. The test used a four-Mecanum-wheeled robot prototype, shown in Figure 13b. The distance between the center lines of the front and rear wheels is 400 mm, and of the left and right wheels is 450 mm. The robot is controlled by another computer with a human–computer interaction (HCI) system. A Hand Rigid Bodies Marker Set was fixed on the robot prototype to test its space coordinates in the test space.

### 4.2. Motion Test System of Mecanum-Wheeled Robot

#### 4.2.1. Motion Shots of the Robot in Test

**Figure 14:**
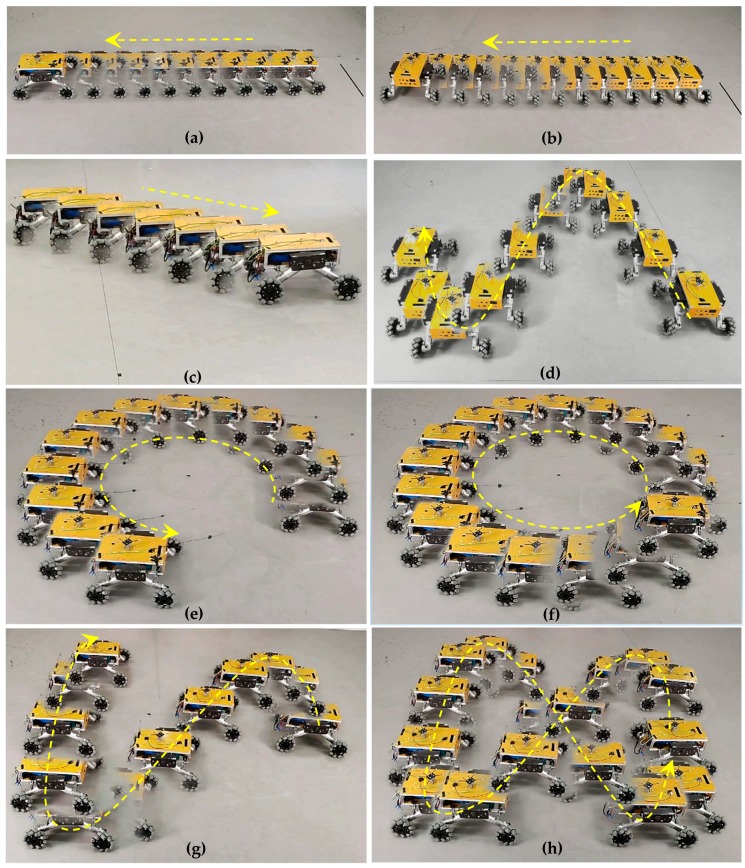
Motion shots of robot in motion test: (**a**) longitudinal translation; (**b**) lateral translation; (**c**) oblique 45° translation; (**d**) translation along a simple harmonic curve; (**e**,**f**) translation along a circle; (**g**,**h**) translation along an 8-shaped curve.

During the testing process, the Mecanum-wheeled robot was controlled to move in these motion modes: longitudinal translation, lateral translation, oblique 45° translation, turning on the spot, circular and 8-shaped curves, and translation motion along a simple harmonic curve. The OptiTrack optical motion capture system captured and recorded the spatial coordinates of the robot. In order to visualize the movement process, the Motion Shot app was used to create a motion picture of a series of coherent images of the robot in the same test environment. The motion picture recorded the position of the robot during movement and showed its movement track. Figure 14 shows six motion pictures of the robot in motion, including longitudinal translation (Figure 14a), lateral translation (Figure 14b), oblique 45° translation (Figure 14c), translation along a circle (Figure 14e,f), 8-shaped curve (Figure 14g,h), and translation along a simple harmonic curve (Figure 14d). The arrows in Figure 14 indicate the moving direction of the robot. 

#### 4.2.2. Analysis and Motion Tests of Robot in Four Simple Motions

(1) Longitudinal and Lateral Motion Tests

**Figure 15:**
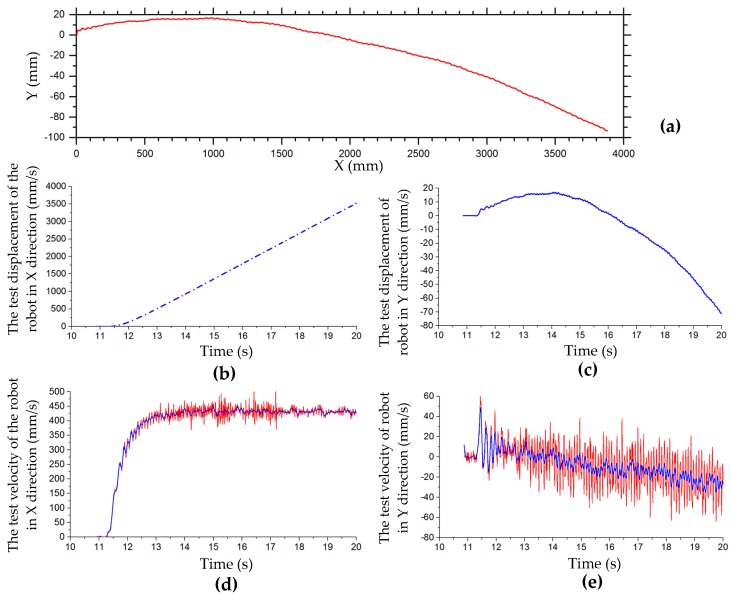
Trajectory, displacement, and velocity curves of robot in lateral translation motion test at a set velocity of 500 mm/s: (**a**) trajectory in lateral translation test; (**b**) lateral displacement curve in x-direction; (**c**) longitudinal displacement curve in y-direction; (**d**) test velocity curve in x-direction; (**e**) test velocity curve in y-direction.

Regardless of the longitudinal or lateral translational motion, there is a certain velocity error of the robot, so the actual velocity is less than the set value. The test velocities and velocity errors are shown in Table 7. When the moving velocity of the robot is set to 100 mm/s, the longitudinal velocity error is 2.18% and the lateral translation velocity error is 12.38%. It can be seen that the lateral movement velocity error is much larger than the longitudinal. The lateral movement test was performed at different set velocities. It can be seen from the test results that the velocity error increased as the set velocity value increased, as shown in Table 7. The loss of velocity during the movement will have a great impact on the trajectory of the robot. Figure 15 shows the trajectory, displacement, and velocity curves of the robot in lateral translation motion test at a set velocity of 500 mm/s.

Figure 15a shows the trajectory of the robot in the lateral translation test at a set velocity of 500 mm/s. The robot moves laterally 3800 mm in the x-axis direction and offset 100 mm in the y-axis direction. The first section of the x-direction velocity curve is an acceleration process; when the set value is reached, the velocity fluctuates at a certain value, as shown in Figure 15d. The lateral displacement curve in the x-direction is shown in Figure 15b. The test displacement and velocity curve of the robot in the y-direction in lateral translation motion is shown in Figure 15c,e, respectively. In the process of lateral translation, in the initial stage, the robot slides to one side, then changes direction, and the sliding displacement accumulates continuously. The velocity curve of the robot along the y-axis fluctuates around the zero line, resulting in a small fluctuation in the trajectory, shown in Figure 15a. 

(2) Test of Oblique Motion at 45°

**Figure 16:**
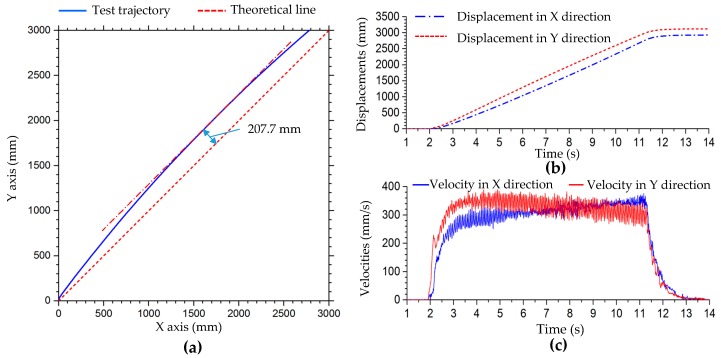
Oblique 45° translation motion test: (**a**) trajectories of robot in oblique 45° translation; (**b**) test displacement curves of the robot in x- and y-directions; (**c**) test velocity curves of the robot in x- and y-directions.

Figure 16a shows the test trajectory of the robot in oblique 45° translation, and Figure 16b,c show the displacement and velocity curves of the robot in the x- and y-directions in oblique 45° translation. In the first half of the 45° oblique motion, the velocity in the y-axis direction is faster than that in the x-axis direction. In the second half of the motion, the velocity in the y-axis direction decreases gradually and is less than that in the x-axis direction. The trajectory of the robot deviates from the theoretical trajectory line in the positive direction of the y-axis, and the second half of the robot gradually approaches the theoretical trajectory line. The trajectory is not a straight line, and there is a certain radian. The maximum distance of the trajectory deviating from the theoretical straight line is 207.7 mm. In this test, the set velocity of the robot is 500 mm/s and the theoretical velocity of the x- and y-directions should be 353.6 mm/s. The average test velocities of the x- and y-axes in the steady state are 319.4 mm/s and 330.7 mm/s, respectively, and the relative errors are −9.67% and −6.47%, respectively.

(3) Test of Motion of Turning on the Spot

**Figure 17:**
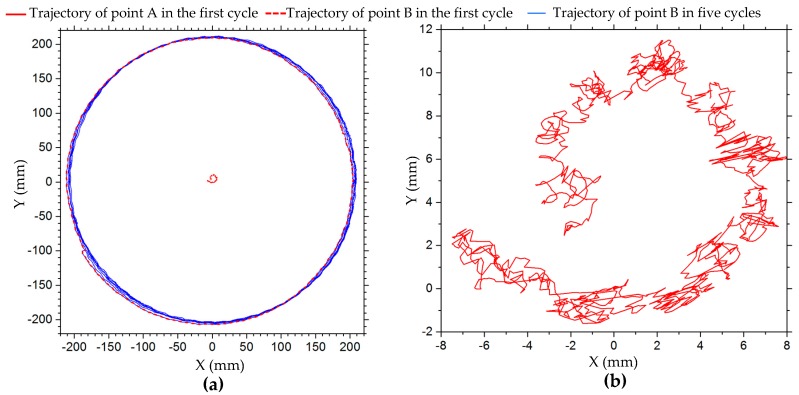
Trajectories of points A and B of the robot in turning on spot; point A is very close to the center, point B is about 200 mm from the center: (**a**) trajectories of point A and B in the first cycle, and the trajectory of point B in five cycles; (**b**) enlarged view of trajectory of point A in the first cycle in (a).

Figure 17 shows the trajectories of points A and B on the robot in turning on the spot, where point A is very close to the center and point B is about 200 mm from the center. Figure 17a shows the trajectories of points A and B in the first cycle, and the trajectory of point B in five cycles. Figure 17b is an enlarged view of the trajectory of point A in the first cycle in Figure 17a. While the robot rotates around one circle, point B draws a perfect circular curve. There is local tortuosity in the circular curve, which indicates that the position of the geometric center of the robot slips during its rotation. The trajectories of point B of multiple rotation cycles do not coincide, and they constitute a trajectory band. By marking and drawing the trajectory of the center point of the robot, we can observe the change of the center point more directly in the course of rotation, but it is difficult to mark accurately. Marker point A can only be located at the geometric center of the robot as far as possible. Marker point B drifts in a small range, and the trajectory of point B in one cycle is shown in Figure 17b, which indicates the displacement variation of the center point of the robot in the process of rotation, and also reflects the trend of circular trajectory caused by the small deviation from the center point (about 3 mm). Table 8 shows the loss and relative errors of the angular velocity of the robot during turning on the spot. This angular velocity error cannot be ignored in precise motion control.

#### 4.2.3. Velocity Compensation in Motion Test of Complex Trajectories

**Figure 18:**
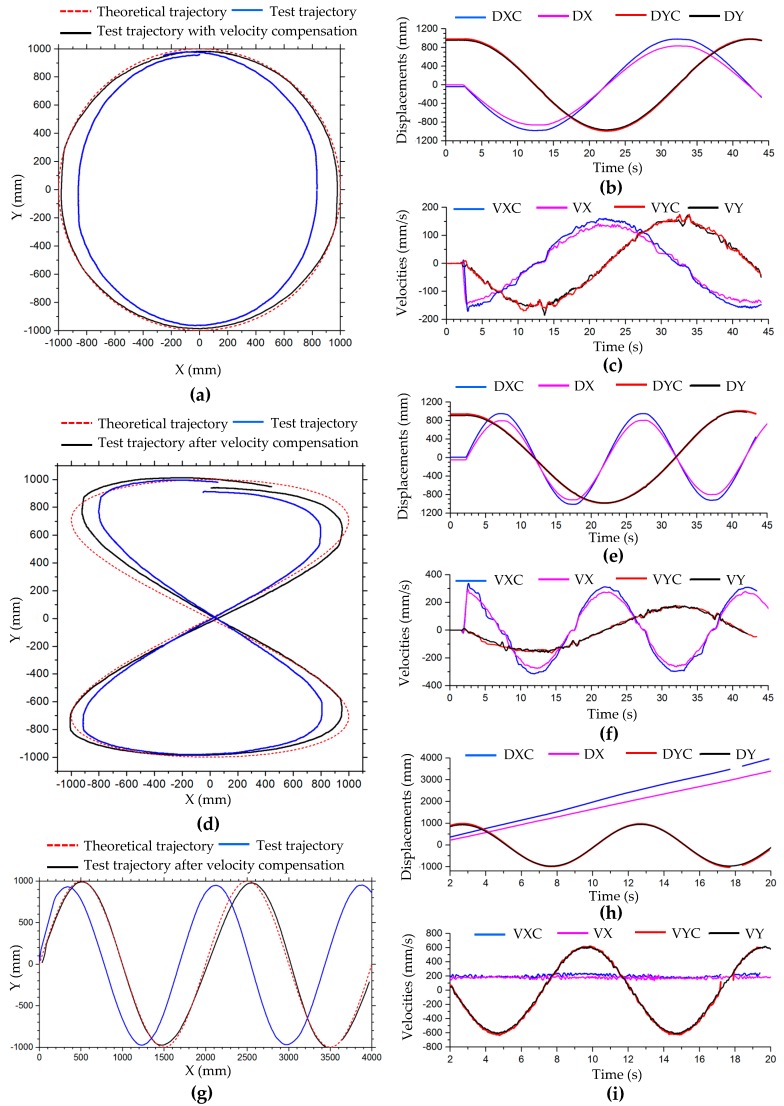
Trajectories, displacements, and velocities, respectively, of the robot in translation motion along (**a**–**c**) a circle, (**d**–**f**) an 8-shaped curve, and (**g**–**i**) a simple harmonic curve. DX or DY, displacement in x- or y-direction; DXC or DYC, displacement in x- or y-direction with velocity compensation; VX or VY, velocity in x- or y-direction; VXC or VYC, velocity in x- or y-direction with velocity compensation.

Figure 18 shows trajectories of translation motion along circle, 8-shaped, and simple harmonic curves. According to the curve equations in Table 2, in the tests, the parameters of the circle curve are r=1000 mm, ω=π/10; the parameters of the 8-shaped curve are l=1000 mm and ω=π/10; and the parameters of the simple harmonic curve are a=300 mm, b=1000 mm and ω=π/5. According to Table 7, the compensation coefficients of longitudinal and lateral velocity are set to 1.04 and 1.145, respectively. Then, the compensation matrix of velocity is set to C_3_.

$$C_{3}=\left[\begin{array}{ccc} 1.145 & 0 & 0\\ 0 & 1.04 & 0\\ 0 & 0 & 0 \end{array}\right]$$

The blue curve in Figure 18a is the test trajectory of the robot in translation motion along a circle, which shifts to the inside of the theoretical circle (dashed red line) and is a vertical approximate ellipse. During the movement, the robot slips or its heading angle changes, causing the trajectory to fail to coincide. After motion compensation, the trajectory of the robot (solid black line) is very consistent with the theoretical curve, as shown in Figure 18a. From the velocity comparison curves (Figure 18c) and displacement comparison curves (Figure 18b) in the x- and y-directions, we can see that velocity and displacement in the motion test with velocity compensation at the same time are greater than the uncompensated velocity and displacement. In the test of translation motion along an 8-shaped curve, the test motion trajectory also deviates greatly from the theoretical trajectory. In the lateral direction, the displacement of the robot is obviously less than the theoretical value, as shown in Figure 18d. In the process of motion, the robot slips and the heading angle changes. Therefore, the test trajectory of the robot deflects. In the first motion cycle, the initial point and termination point of the robot cannot coincide. The displacements and velocities in translation motion along an 8-shaped curve are shown in Figure 18d,f, respectively. In the test of translational motion of a simple harmonic curve, the error of longitudinal displacement (x-axis direction) is small, while that of transverse displacement (y-axis direction) is large. With the increased motion cycles of the robot, the deviation between transverse displacement and theoretical trajectory curve increases, as shown in Figure 18g. This is consistent with the trend of dynamic simulation. The robot maintains a uniform velocity in the x-direction, and the speed in the x-direction with compensation (solid blue line) is slightly larger, as shown in Figure 18i; the displacement curve in the x-direction with motion compensation is at the top, as shown in Figure 18h. In the y-direction, the velocity and displacement of the robot change periodically, and are compensated accordingly. Table 9 shows the relative errors of displacement in motion simulation along a circle, 8-shaped curve, and simple harmonic curve in Figure 18. From Table 9, improvement of the robot’s displacement accuracy using the kinematic model with velocity compensation can be observed more clearly.

From the results of the motion compensation test, we can get the following conclusions: (1) in the real environment, the wheel slippage of a Mecanum-wheeled robot has a great impact on the robot’s motion accuracy, especially lateral motion; (2) for motion with small velocity change, the constant velocity compensation matrix can have a good compensation effect, and the actual trajectories of the robot are very consistent with the theoretical curves; (3) more accurate motion compensation requires the use of a variable velocity compensation matrix, which is dynamically set according to the robot’s velocity; (4) in the process of motion, the robot will deviate from its heading, which will cause its trajectory to deviate; and (5) according to the robot’s parameters and environment information detected by position, attitude, and speed sensors, the motion direction and velocity can be adjusted in real time by using this kinematic model with speed compensation, which is more conducive to the robot’s motion accuracy.

## 5. Motion Simulation of Combined Configurations of Multiple Four-Mecanum-Wheeled Robots 

In Section 3, the typical motions of the four-Mecanum-wheeled robot were simulated. In Section 4, motion tests were performed using the robot physical prototype. Although the geometry of the virtual prototype used for motion simulation and the physical prototype used for testing are different, the motion test results verify the feasibility and effectiveness of motion simulation of Mecanum-wheeled robots by RecurDyn software. Therefore, the motion model of combined configurations of multiple-Mecanum-wheeled robots can also be verified by this simulation method, which can also make up for the shortcomings that we only have one physical robot sample and cannot achieve a collaborative combined multiple-robot experiment. In this section, kinematic simulations of a variety of configurations are performed to verify the kinematic model of multiple robots. According to the motion simulations in Section 3, the relative error between the simulation result and the theoretical value is small. Even without motion compensation, the simulation trajectory of the robot is very close to the theoretical curve. Therefore, in this section, in order to carry out simulations efficiently, simulations without motion compensation are first carried out for various combination configurations, and then two robot combination configurations are selected for simulation with motion compensation.

### 5.1. Multiple Four-Mecanum-Wheeled Robot Combination Configurations for Simulation

**Figure 19:**
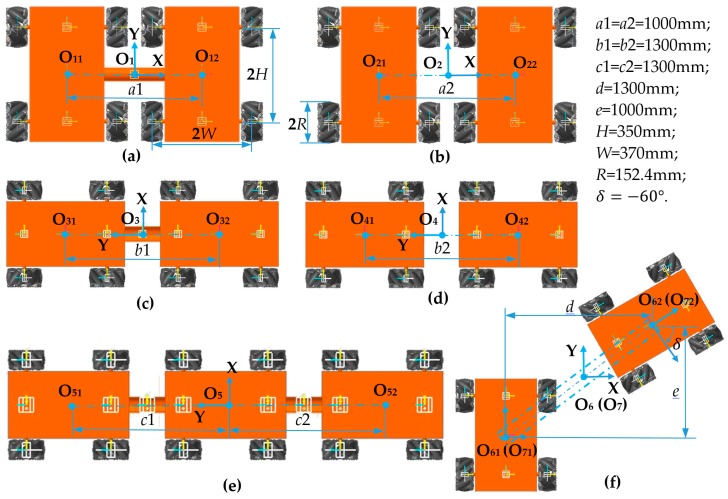
Configurations of multiple four-Mecanum-wheeled robots: (**a**) side-by-side connected configuration of two robots (SCC-2); (**b**) side-by-side unconnected configuration of two robots (SUC-2); (**c**) tandem connected configuration of two robots (TCC-2); (**d**) tandem unconnected configuration of two robots (TUC-2); (**e**) tandem connected configuration of three robots (TCC-3); (**f**) arbitrary unconnected configuration of two robots (AUC-2); if the two robots are connected, it is an arbitrary connected configuration (ACC-2).

In this section, seven multiple robot combination configurations are used for motion simulation. The configurations and their main dimensions are shown in Figure 19. Figure 19a,b show two four- Mecanum-wheeled robots arranged laterally. The center distances of the two robots are the same; the two robots in Figure 19a are connected in the middle, called side-by-side connected configuration of two robots (SCC-2), and the two in Figure 19b are separate, called side-by-side unconnected configuration of two robots (SUC-2). Figure 19c,d show longitudinally arranged dual four-Mecanum-wheeled robots with the same center-to-center distance. The two robots in Figure 19c are connected, called tandem connected configuration of two robots (TCC-2), and the two in Figure 19d are separate, called tandem unconnected configuration of two robots (TUC-2). Figure 19e shows a tandem connected configuration of three robots (TCC-3). Figure 19f shows an arbitrary unconnected configuration of two robots arranged at an angle of 30° (AUC-2). If the two robots are connected, this is an arbitrary connected configuration (ACC-2).

### 5.2. Motion Simulation of Configurations of Multiple Four-Mecanum-Wheeled Robots 

#### 5.2.1. Simulation of Turning on Spot of Multiple Four-Mecanum-Wheeled Robots

**Figure 20:**
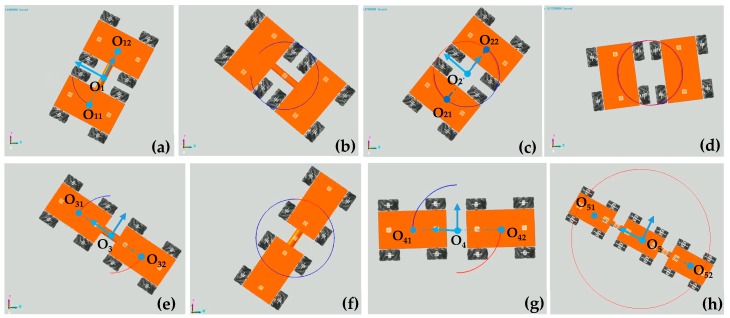
Simulation of turning on the spot of multiple four-Mecanum-wheeled Robots: (**a**,**b**) SCC-2; (**c**,**d**) SUC-2; (**e**,**f**) TCC-2; (**g**) TUC-2; (**h**) TCC-3.

**Figure 21:**
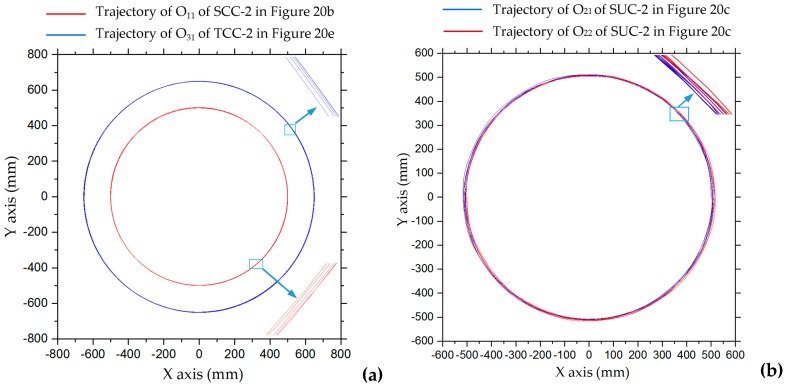
Trajectories of points on the robots in simulation of turning on the spot after five cycles: (**a**) O_11_ of SCC-2 in Figure 20b and O_31_ of TCC-2 in Figure 20e; (**b**) O_21_ and O_22_ of SUC-2 in Figure 20c.

**Figure 22:**
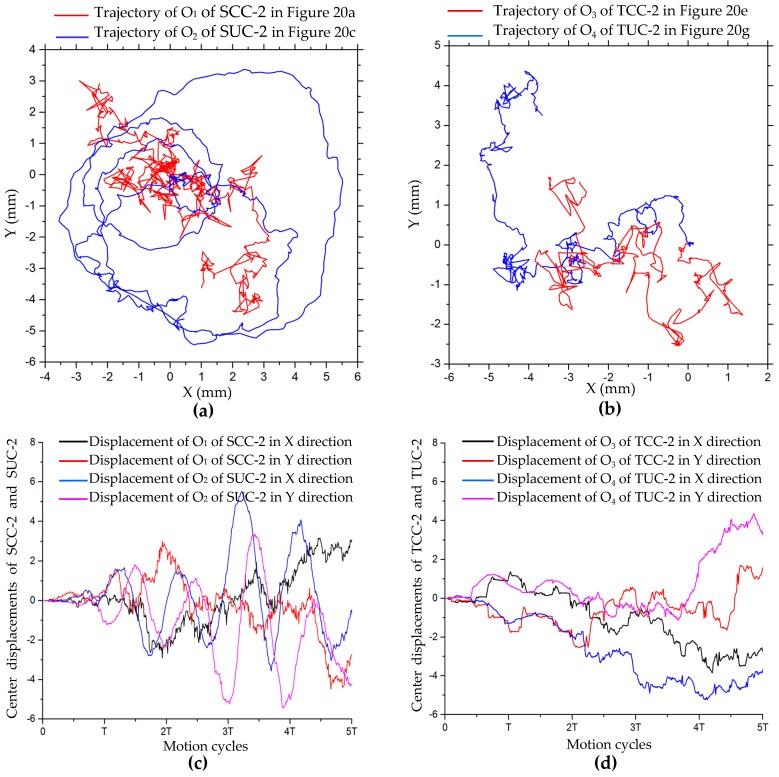
Trajectories of robot centers and center offset curves for in situ rotating motion: (**a**) centers O_1_ of SCC-2 and O_2_ of SUC-2; (**b**) centers O_3_ of TCC-2 and O_4_ of TUC-2; (**c**) displacement curves of SCC-2 and SUC-2 in x and y directions; (**d**) displacement curves of TCC-2 and TUC-2 in x- and y-directions.

In situ rotation motion simulation of the five configurations of robots was carried out, as shown in Figure 20. Figure 21 shows trajectories of the points on the robots in the simulation of turning on the spot after five cycles. The trajectories of O_11_ of SCC-2 in Figure 20b and of O_31_ of TCC-2 in Figure 20e are shown in Figure 21a; Figure 21b shows trajectories of O_21_ and O_22_ of SCC-2 in Figure 20c. We can see that after the robot rotates in place for many cycles, the trajectory of the marker point on the robot forms a circular trajectory range. In comparison, the trajectories of two unconnected robots (SUC-2) are relatively tortuous, as shown in Figure 21b. This is because the robots randomly move in the plane during in situ rotation. Figure 22 shows the trajectories of the center points of the combined robots and displacement curves in both x- and y-directions during the rotation. The center trajectory coverage of the unconnected robots is larger than that of the connected robots, as shown in Figure 22a,b, and their centers also have larger displacements in the x- or y-direction, as shown in Figure 22c,d. With rotation, there are certain periodic variations in displacement, and displacement has an increasing tendency, as shown in Figure 22c,d.

#### 5.2.2. Translation Simulation along a Circle of Multiple Four-Mecanum-Wheeled Robots

**Figure 23:**
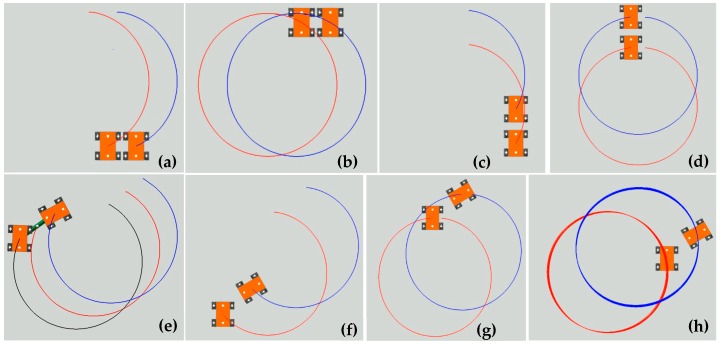
Translation simulation process along a circle: (**a**,**b**) SUC-2; (**c**,**d**) TUC-2; (**e**) ACC-2; (**f**,**g**) AUC-2; (**h**) the state of AUC-2 after multiple rotation cycles.

**Figure 24:**
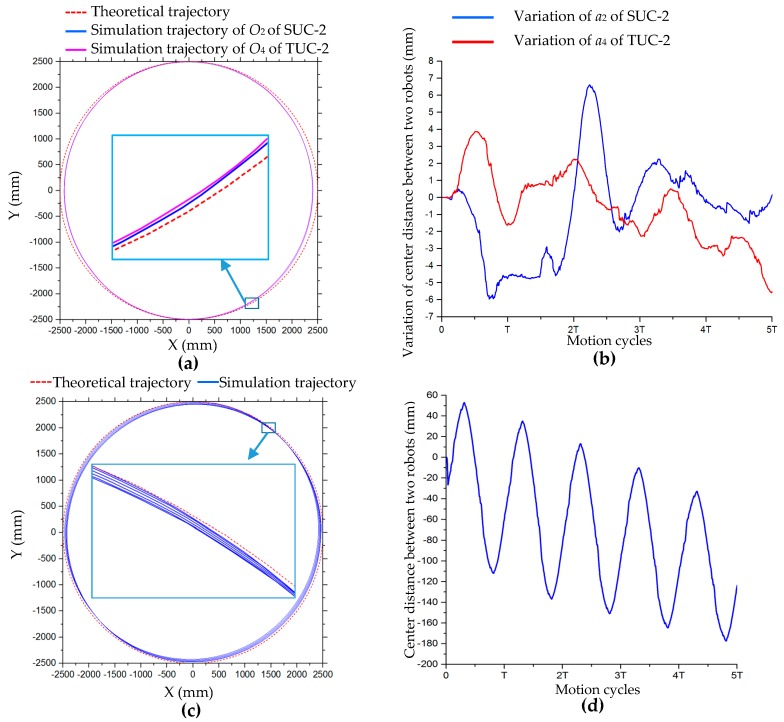
Trajectories of centers and variation curves of center distance between two robots in translation simulation along a circle: (**a**) trajectories of centers of SUC-2 and TUC-2; (**b**) variation curves of center distance between two robots of SUC-2 and TUC-2; (**c**) simulation trajectory of center *O*_6_ of AUC-2; (**d**) variation curve of center distance between two robots of AUC-2.

Three robot configurations, SUC-2, TUC-2, and AUC-2, were simulated for translational motion around a circle with a radius of 2500 mm. The simulation process, shown in Figure 23, verifies the correctness of the motion model of multiple-robot combination configurations. Figure 24a shows the trajectories of the centers O_2_ of SUC-2 and O_4_ of TUC-2; these two trajectories are very close, but because the simulation does not perform velocity compensation, they are smaller than the theoretical circle in the x-direction. In the process of simulation, the distance between the two robots in two configurations, SUC-2 and TUC-2, changes. The change curves of the distance between the two robots in these two configurations in the simulation process are shown in Figure 24b, which shows that the relative positions of the two robots change. From Figure 24c, it can be seen that the trajectory of center O_6_ of configuration AUC-2 changes greatly during several motion cycles, and the change of distance between the two robots in this configuration is relatively large and has certain periodicity, as shown in Figure 24d.

#### 5.2.3. Translation Simulation along an 8-Shaped Curve of Multiple Four-Mecanum-Wheeled Robots

**Figure 25:**
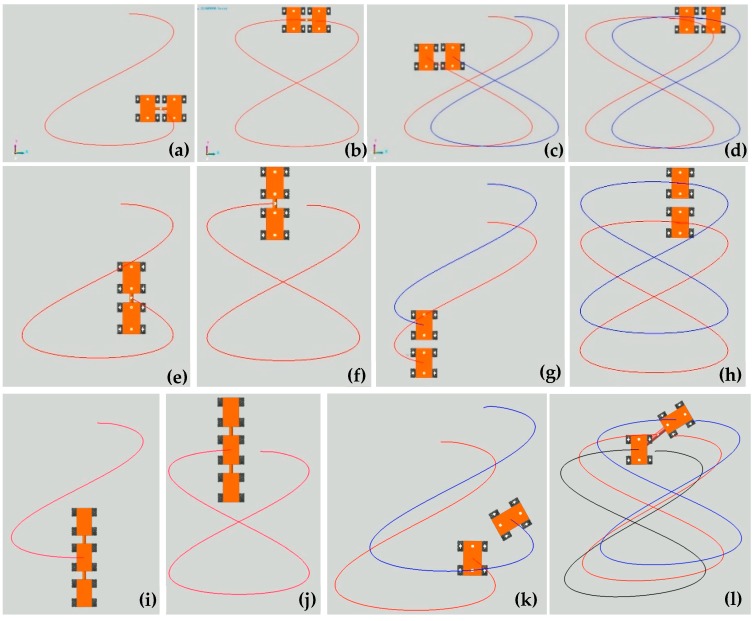
Translation motion simulation process of the seven configurations in Figure 19 along an 8-shaped curve: (**a**,**b**) SCC-2; (**c**,**d**) SUC-2; (**e**,**f**) TCC-2; (**g**,**h**) TUC-2; (**i**,**j**) TCC-3; (**k**) AUC-2; (**l**) ACC-2.

**Figure 26:**
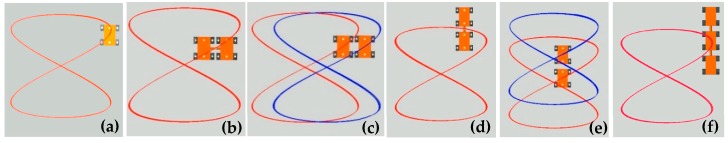
State of four configurations in translation motion along an 8-shaped curve after several cycles: (**a**) single four-Mecanum-wheeled robot; (**b**) SCC-2; (**c**) SUC-2; (**d**) TCC-2; (**e**) TUC-2; (**f**) TCC-3.

**Figure 27:**
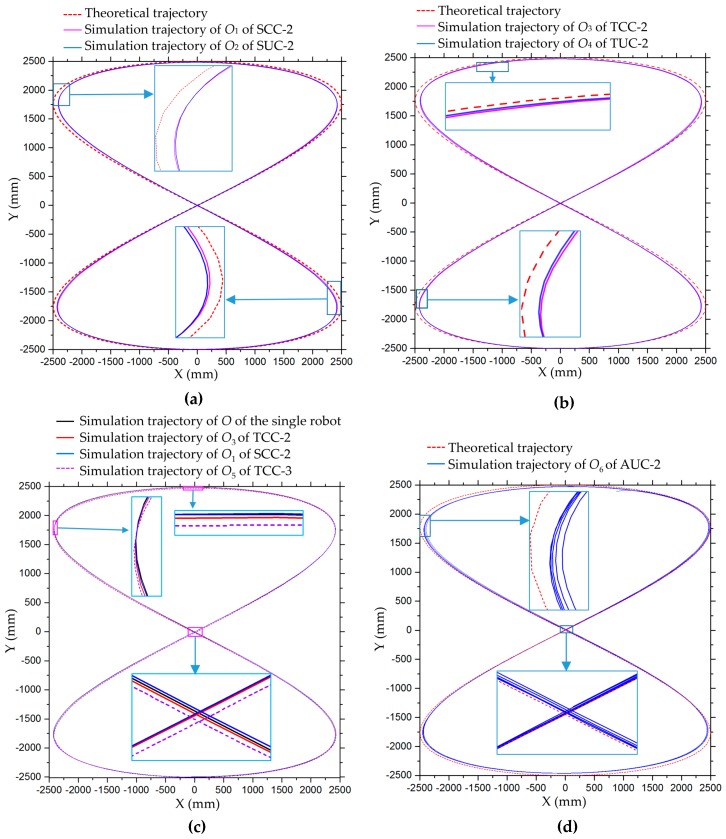
Trajectories of the centers of the robots in translation simulation along an 8-shaped curve: (**a**) centers *O*_1_ of SCC-2 and *O*_2_ of SUC-2; (**b**) centers *O*_3_ of TCC-2 and *O*_4_ of TUC-2; (**c**) multiple cycles trajectory of *O*_6_ on AUC-2; (**d**) simulation trajectories of a single robot, TCC-2, SCC-2 and TCC-3.

Figure 25 shows the translation motion simulation process of the seven combination configurations in Figure 19 along an 8-shaped curve, which is defined in Table 2. Figure 26 shows the state after these robots have moved for several cycles. In Figure 27, the motion trajectories of these simulations are compared. According to Figure 27, since no velocity compensation is performed in the motion simulations, there is obvious displacement error in the x-direction for all simulation trajectories. For the combination of two robots arranged side-by-side or end-to-end, the error of their motion trajectories in the first cycle is similar whether they are connected or not, as shown in Figure 27a,b. For the combination of connected multiple robots, the error of the simulation track does not show an obvious difference, as shown in Figure 27c. After several cycles of motion, the trajectory of each moving robot will shift to a certain extent, and these trajectories form a trajectory band, as shown in Figure 26. Taking AUC-2 as an example, the trajectory band after five cycles of robot combination rotation is shown in Figure 27d.

#### 5.2.4. Translation Simulation along Simple Harmonic Motion Curve of the Configurations of Multiple Four-Mecanum-Wheeled Robots

**Figure 28:**
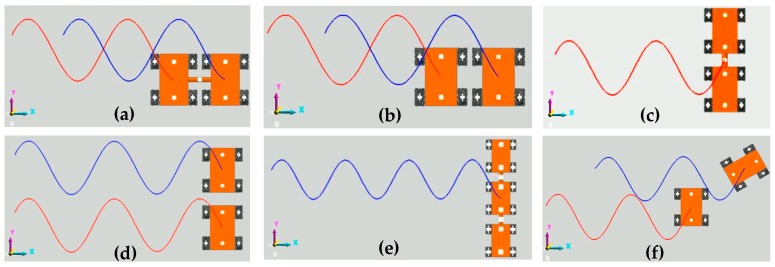
Translation motion simulation process of configurations along a simple harmonic motion curve: (**a**) SCC-2; (**b**) SUC-2; (**c**) TCC-2; (**d**) TUC-2; (**e**) TCC-3; (**f**) AUC-2.

**Figure 29:**
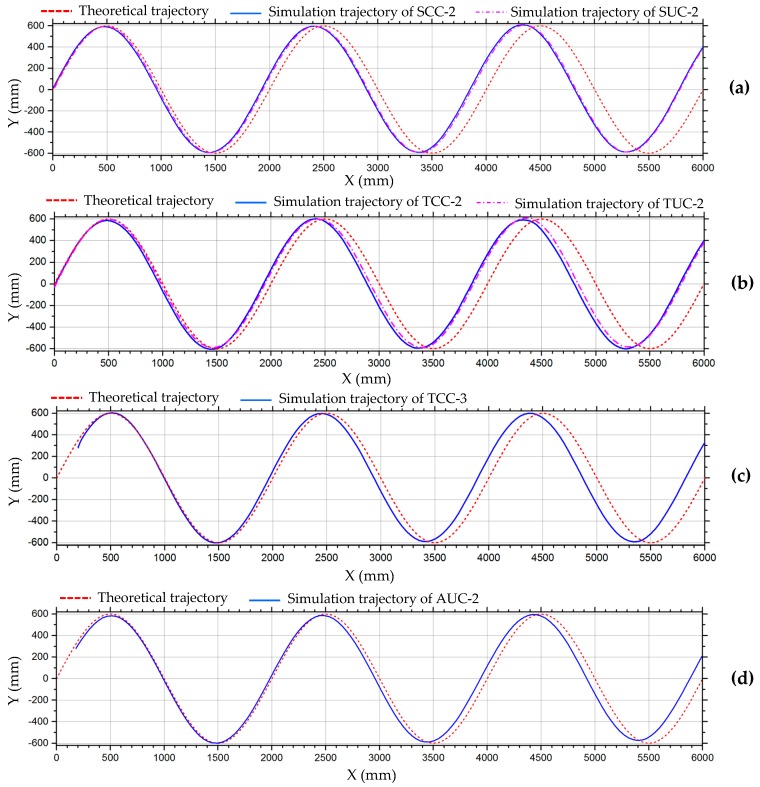
Simulation trajectories of the six configurations in Figure 19 along a simple harmonic motion curve: (**a**) O_1_ of SCC-2 and O_2_ of SUC-2; (**b**) O_3_ of TCC-2 and O_4_ of TUC-2; (**c**) O_5_ of TCC-3; (**d**) O_6_ of AUC-2.

Figure 28 shows the translation motion simulation process of the six combination configurations in Figure 19 along a simple harmonic motion curve, which is defined in Table 2. Figure 29 shows the motion simulation trajectories of the robots. It can be seen that the simulation trajectories are close to the theoretical trajectories in the first motion cycle, but as the motion cycles increase, the error of the robot in the x-direction accumulates, causing the simulation trajectories to lag behind the theoretical trajectories. In the y-direction, trajectory errors are not accumulated, and because the longitudinal velocity errors are small, the displacements of the robots in the y-direction can almost reach the theoretical value.

### 5.3. Motion Simulation after Velocity Compensation for TCC-3

**Figure 30:**
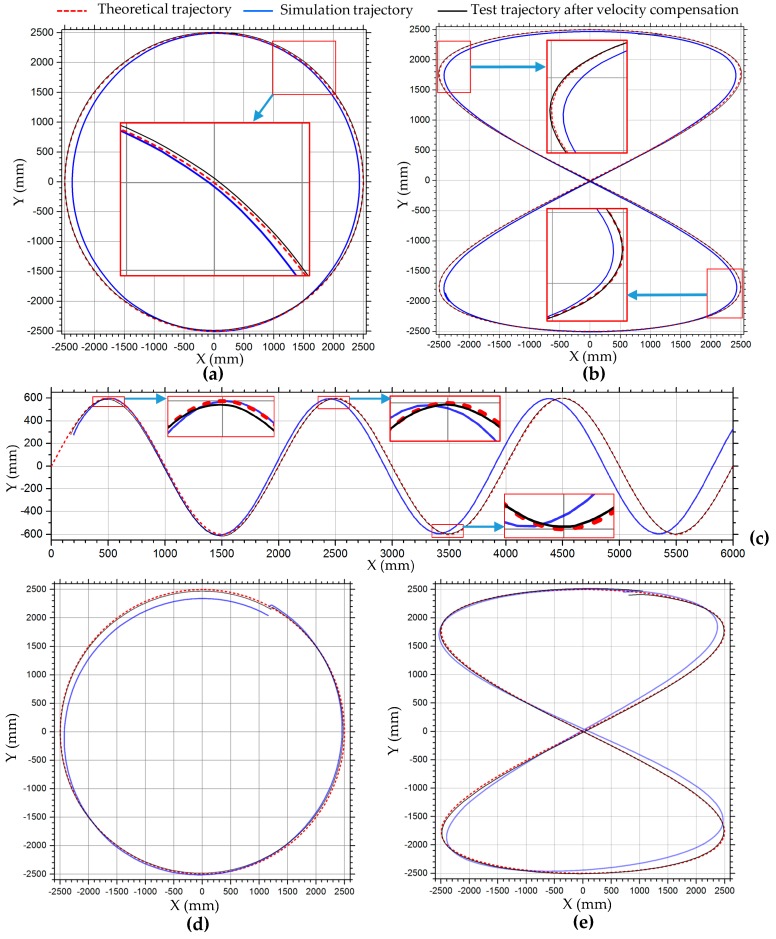
Trajectories of O_5_ of TCC-3 and O_7_ of ACC-2 in motion simulation: (**a**) circular trajectories of TCC-3; (**b**) 8-shaped trajectories of TCC-3; (**c**) simple harmonic motion trajectories of TCC-3; (**d**) circular trajectories of ACC-2; (**e**) 8-shaped trajectories of ACC-2.

In Section 5.2, the motion simulations of the combination configurations of robot were carried out, and the kinematic model of the multiple-robot combination was verified. However, in the simulation experiment, there was no velocity compensation, so there were obvious errors between the robot’s trajectories and the theoretical curves, especially in the x-direction of the local coordinate system. In this section, the robot configuration of TCC-3 and ACC-2 are selected and motion compensation simulation is carried out to verify the applicability of the velocity compensation model of multiple robot combination configurations. The velocity compensation coefficients used in this simulation are also obtained through the longitudinal and lateral translation motion simulation. In Figure 30, compared with the simulation curves without velocity compensation drawn by solid blue lines, the trajectories of the robot after compensation, drawn by solid black lines, are significantly improved, and are very consistent with the theoretical curves drawn by the dashed red lines.

## 6. Conclusions

In order to solve the problem of transporting large-scale goods or equipment in industry, a combination system of multiple Mecanum wheels or multiple-Mecanum-wheeled robots is usually adopted. The kinematics of this combined mobile system is the theoretical basis for the motion control, path planning, and navigation of the combined system. In a real environment, slippage of Mecanum wheels has a great impact on the robot’s motion accuracy, especially lateral motion. In this work, based on studying the kinematic constraints of a single Mecanum wheel in a mobile system, a kinematic model of Mecanum-wheeled robots with multiple wheels is derived, then a kinematic model with velocity compensation of a combination mobile system composed of multiple Mecanum-wheeled robots is created. Taking four-Mecanum-wheeled robots as example, motion simulations of a virtual prototype on RecurDyn and motion tests of a physical prototype were carried out to verify the kinematic model of the robot and the motion compensation model. For motion with small velocity change, the constant velocity compensation matrix has a good compensation effect, and the actual trajectories of the robot are very consistent with the theoretical curves. Finally, the motions of a variety of combined mobile systems composed of multiple Mecanum-wheeled robots were simulated on RecurDyn. Motion simulations and tests prove that the kinematic model of single robot and multi-robot combined mobile systems is correct, and the inverse kinematic correction model with velocity compensation matrix is feasible. Through simulation or experiment, the velocity compensation coefficient of the robots can be obtained and the velocity compensation matrix can be created. This modified inverse kinematic model can effectively reduce the errors of motion caused by wheel slippage and improve the motion accuracy of the robot mobile system.

More accurate motion compensation requires the use of a variable velocity compensation matrix, which is dynamically set according to the robot’s velocity. According to the robot’s parameters and environment information detected by position, attitude, and speed sensors, the robot’s motion direction and velocity can be adjusted in real time by using this kinematic model with speed compensation, which is more conducive to motion accuracy.

## Figures and Tables

**Table 1:** Material property parameters of ground and roller.

Material	Elastic Modulus E (MPa)	Poisson’s Ratio *υ*
Concrete	30,000	0.20
Polyurethane	90	0.45

**Table 2:** Trajectory equations of robot moving in circular, figure-8-shaped, and harmonic curves.

Names of Curve	Trajectory Equations in Figure 6
Circular curve	$x=r_{i}\sin\omega_{i}t,\;y=r_{i}\cos\omega_{i}t,\;\theta =0$, where $r_{1}=2500\;\mathrm{mm}$, $r_{2}=r_{3}=1350\;\mathrm{mm}$, $\omega_{1}=\pi /10$, $\omega_{2}=\omega_{3}=\pi /5$
Figure-8-shaped curve	x=lsin2ωt, y=lcosωt, θ=0, where l=2500 mm, ω=π/10
Harmonic curve	x=at, y=bsinωt, θ=0, where a=400 mm/s, b=600 mm, ω=2π/5

**Table 3:** Velocity errors and compensation coefficients in longitudinal and lateral motion simulation.

Motion Mode	Set Velocity Value (mm/s)	Average Simulation Velocity (mm/s)	Relative Error (%)	Compensation Coefficient	Average Compensation Coefficient
Longitudinal translation motion	100	99.767	−0.233	1.00234	1.002
	200	199.564	−0.218	1.00218	
	300	299.339	−0.220	1.00221	
	400	399.151	−0.212	1.00212	
	500	498.905	−0.219	1.00219	
Lateral translation motion	100	96.928	−3.072	1.03169	1.033
	200	193.684	−3.158	1.03261	
	300	290.504	−3.165	1.03269	
	400	386.930	−3.268	1.03378	
	500	483.158	−3.368	1.03486	

**Table 4:** Velocity error of simulation results.

Oblique 45° Motion	Set Velocity Value (mm/s)	Average Simulation Velocity in Steady State (mm/s)	Relative Error (%)
X- axis direction	200	196.566	−1.717
Y- axis direction	200	196.65	−1.675
X- axis direction	500	490.061	−1.988
Y- axis direction	500	490.164	−1.967

**Table 5:** Relative error of angular velocity in simulation of turning on the spot.

Set Angular Velocity Value (rad/s)	Average Angular Velocity (rad/s)	Relative Error (%)	Compensation Coefficient
$\frac{\pi }{12}$ (=0.262)	0.257	−1.753	1.0195
$\frac{\pi }{6}$ (=0.524)	0.514	−1.920	1.0195
$\frac{\pi }{4}$ (=0.785)	0.768	−2.182	1.0221

**Table 6:** Relative errors of displacement in motion simulation along circular and 8-shaped curves.

Motion Mode		Maximum Displacement (mm)	Minimum Displacement (mm)	Relative Error (%)
Circular motion in Figure 12a	X-axis	2430.51	−2395.56	−3.48
	Y-axis	2494.11	−2498.98	−0.14
8-shaped curve motion in Figure 12b	X-axis	2423.55	−2430.37	−2.92
	Y-axis	2494.90	−2490.90	−0.28

**Table 7:** Velocity errors and compensation coefficients in motion tests.

Motion Mode	Set Velocity Value (mm/s)	Average Test Velocity in Steady State (mm/s)	Relative Error (%)	Compensation Coefficient
Longitudinal translation motion	100	97.822	−2.178	1.022
	200	196.287	−1.857	1.019
	300	293.731	−2.090	1.021
	400	390.558	−2.361	1.024
	500	486.907	−2.619	1.027
Lateral translation motion	100	87.621	−12.379	1.141
	200	174.193	−12.904	1.148
	300	260.675	−13.108	1.151
	400	345.426	−13.644	1.158
	500	432.065	−13.587	1.157

**Table 8:** Relative error of angular velocity in motion test of turning on the spot.

Set Angular Velocity Value (rad/s)	Average Angular Velocity (rad/s)	Relative Error (%)
$\frac{\pi }{6}$ (=0.524)	0.511	−2.550
$\frac{\pi }{3}$ (=1.047)	1.020	−2.467

**Table 9:** Relative errors of displacement in motion test of complex trajectories.

Motion Mode		Dmax (mm)	Dmin (mm)	DCmax (mm)	DCmin (mm)	RED (%)	REDC (%)
Circular motion in Figure 18a	X-axis	834.757	−860.636	978.495	−981.766	−15.23%	−1.99%
	Y-axis	978.196	−964.902	982.437	−987.510	−2.85%	−1.50%
8-shaped curve motion in Figure 18d	X-axis	807.439	−914.31	952.785	−1008.12	−13.91%	−1.95%
	Y-axis	995.717	−980.872	1013.374	−985.743	−1.17%	−0.04%
A simple harmonic curve in Figure 18g	Y-axis	950.934	−974.760	990.842	−1005.300	−3.715%	−0.191%

Note: Dmax, maximum displacement; Dmin, minimum displacement; DCmax, maximum displacement with velocity comparison; DCmin, minimum displacement with velocity comparison; RED, relative error of displacement; REDC, relative error of displacement with velocity comparison.

## References

[1] Dang Q.V., Nguyen C.T., Rudová H. (2018). Scheduling of mobile robots for transportation and manufacturing tasks. J. Heuristics.

[2] Bogh S., Schou C., Rühr T., Kogan Y., Dömel A., Brucker M., Eberst C., Tornese R., Sprunk C., Tipaldi G.D. Integration and assessment of multiple mobile manipulators in a real-world industrial production facility. Proceedings of the 45th International Symposium on Robotics and the 8th German Conference on Robotics (ISR/Robotik 2014).

[3] Chawla V.K., Chanda A.K., Angra S. (2019). The scheduling of automatic guided vehicles for the workload balancing and travel time minimi-zation in the flexible manufacturing system by the nature-inspired algorithm. J. Proj. Manag..

[4] Du L.Z., Ke S.F., Wang Z., Tao J., Yu L.Q., Li H.J. (2019). Research on multi-load AGV path planning of weaving workshop based on time priority. Math. Biosci. Eng..

[5] Chen C., Huy D.T., Tiong L.K., Chen I.-M., Cai Y.Y. (2019). Optimal facility layout planning for AGV-based modular prefabricated manufacturing system. Autom. Constr..

[6] Chen C., Tiong L.K., Chen I.M. (2019). Using a genetic algorithm to schedule the space-constrained AGV-based prefabricated bathroom units manufacturing system. Int. J. Prod. Res..

[7] Dehnavi-Arani S., Saidi-Mehrabad M., Ghezavati V. (2019). An integrated model of cell formation and scheduling problem in a cellular manufacturing system considering automated guided vehicles’ movements. Int. J. Oper. Res..

[8] Qian J., Zi B., Wang D.M., Ma Y.G., Zhang D. (2017). The design and development of an omni-directional mobile robot oriented to an intelligent manufacturing system. Sensors.

[9] Sun S.K., Hu J.P., Li J., Liu R.D., Shu M., Yang Y. (2019). An INS-UWB based collision avoidance system for AGV. Algorithms.

[10] Xie L., Scheifele C., Xu W.L., Stol K.A. Heavy-duty Omni-directional Mecanum-wheeled Robot for Autonomous Navigation: System Development and Simulation Realization. Proceedings of the 2015 IEEE International Conference on Mechatronics (ICM).

[11] Heß D., Künemund F., Röhrig C. Linux based control framework for Mecanum based omnidirectional automated guided vehicles. Proceedings of the World Congress on Engineering and Computer Science 2013.

[12] Han L., Qian H.H., Chung W.K., Hou K.W., Lee K.H., Chen X., Zhang G.H., Xu Y.S. System and design of a compact and heavy-payload AGV system for flexible production line. Proceedings of the 2013 IEEE International Conference on Robotics and Biomimetics (ROBIO).

[13] Zachewicz B. Flugzeugtransporter mecanumbasierend. https://www.youtube.com/watch?v=U1MAv7tz9wM.

[14] Airbus Rolls Out Its Second A350 XWB Composite Fuselage Demonstrator. https://www.airbus.com/newsroom/news/en/2009/08/airbus-rolls-out-its-second-a350-xwb-composite-fuselage-demonstrator.html.

[15] KUKA omniMove. https://www.kuka.com/en-de/products/mobility/mobile-platforms/kuka-omnimove.

[16] Automation K.-R. KUKA omniMove at Siemens plant Krefeld. https://www.youtube.com/watch?v=EvOrFgSmQoc.

[17] Muir P.F., Neuman C.P. (1987). Kinematic modeling of wheeled mobile robots. J. Robot. Syst..

[18] Muir P.F. (1988). Modeling and Control of Wheeled Mobile Robots. Ph.D. Dissertation.

[19] Muir P.F., Neuman C.P. Kinematic modeling for feedback control of an omnidirectional wheeled mobile robot. Proceedings of the 1987 IEEE International Conference on Robotics and Automation.

[20] Campion G., Bastin G., Dandrea-Novel B. (1996). Structural properties and classification of kinematic and dynamic models of wheeled mobile robots. IEEE Trans. Robot. Autom..

[21] Röhrig C., Heß D., Künemund F. Motion controller design for a mecanum wheeled mobile manipulator. Proceedings of the 2017 IEEE Conference on Control Technology and Applications (CCTA).

[22] Tuci E., Alkilabi M.H.M., Akanyeti O. (2018). Cooperative Object Transport in Multi-Robot Systems: A Review of the State-of-the-Art. Front. Robot. AI.

[23] Alonso-Mora J., Knepper R., Siegwart R., Rus D. Local motion planning for collaborative multi-robot manipulation of deformable objects. Proceedings of the 2015 IEEE International Conference on Robotics and Automation (ICRA).

[24] Alonso-Mora J., Baker S., Rus D. (2017). Multi-robot formation control and object transport in dynamic environments via constrained optimization. Int. J. Robot. Res..

[25] Habibi G., Kingston Z., Xie W., Jellins M., McLurkin J. Distributed centroid estimation and motion controllers for collective transport by multirobot systems. Proceedings of the 2015 IEEE International Conference on Robotics and Automation (ICRA).

[26] Habibi G., Xie W., Jellins M., McLurkin J. (2016). Distributed path planning for collective transport using homogeneous multi-robot systems. Distributed Autonomous Robotic Systems.

[27] Lippi M., Marino A. Cooperative object transportation by multiple ground and aerial vehicles: Modeling and planning. Proceedings of the 2018 IEEE International Conference on Robotics and Automation (ICRA).

[28] Verginis C.K., Nikou A., Dimarogonas D.V. (2018). Communication-based decentralized cooperative object transportation using nonlinear model predictive control. arXiv.

[29] Tsai C.C., Wu H.L., Tai F.C., Chen Y.S. Decentralized cooperative transportation with obstacle avoidance using fuzzy wavelet neural networks for uncertain networked omnidirectional multi-robots. Proceedings of the 2016 12th IEEE International Conference on Control and Automation (ICCA).

[30] Wang Z., Yang G., Su X.S., Schwager M. (2018). OuijaBots: Omnidirectional robots for cooperative object transport with rotation control using no communication. Distributed Autonomous Robotic Systems.

[31] Paniagua-Contro P., Hernandez-Martinez E.G., González-Medina O., González-Sierra J., Flores-Godoy J.J., Ferreira-Vazquez E.D., Fernandez-Anaya G. (2019). Extension of Leader-Follower Behaviours for Wheeled Mobile Robots in Multirobot Coordination. Math. Probl. Eng..

[32] Chu B. (2017). Position Compensation Algorithm for Omnidirectional Mobile Robots and Its Experimental Evaluation. Int. J. Precis. Eng. Manuf..

[33] Kulkarni S.G., Mulay G.N., Patil T., Parkhe C. (2015). Automated High Speed Omnidirectional Navigation Using Closed Loop Implementation of Four Wheel Holonomic Mecanum Drive. Spvryan’s Int. J. Eng. Sci. Technol. (SEST).

[34] Tian P., Zhang Y.N., Zhang J., Yan N.M., Zeng W. (2013). Research on Simulation of Motion Compensation for 8 × 8 Omnidirectional Platform Based on Back Propagation Network. Appl. Mech. Mater..

[35] Udomsaksenee J., Wicaksono H., Nilkhamhang I. Glocal Control for Mecanum-Wheeled Vehicle with Slip Compensation. Proceedings of the 2018 15th International Conference on Electrical Engineering/Electronics, Computer, Telecommunications and Information Technology (ECTI-CON).

[36] Keek J.S., Loh S.L., Chong S.H. (2019). Comprehensive Development and Control of a Path-Trackable Mecanum-Wheeled Robot. IEEE Access.

[37] Adamov B.I. (2018). A Study of the Controlled Motion of a Four-wheeled Mecanum Platform. Russ. J. Nonlinear Dyn..

[38] Wen R.Y., Tong M.M. Mecanum wheels with Astar algorithm and fuzzy PID algorithm based on genetic algorithm. Proceedings of the 2017 International Conference on Robotics and Automation Sciences (ICRAS).

[39] Kim J., Woo S., Kim J., Do J., Kim S., Bae S. (2012). Inertial navigation system for an automatic guided vehicle with Mecanum wheels. Int. J. Precis. Eng. Manuf..

[40] Li Y.W., Dai S.M., Zhao L.L., Yan X.C., Shi Y. (2019). Topological Design Methods for Mecanum Wheel Configurations of an Omnidirectional Mobile Robot. Symmetry.

[41] Wang Y.Z., Chang D.G. (2009). Motion Performance Analysis and Layout Selection for Motion System with Four Mecanum Wheels. J. Mech. Eng..

[42] Wang Y.Z., Chang D.G. (2009). Motion restricted condition and singular configuration for mecanum wheeled omni-directional motion system. J. Shanghai Univ. (Nat. Sci.).

[43] Lin L.-C., Shih H.-Y. (2013). Modeling and adaptive control of an omni-Mecanum-wheeled robot. Intell. Control Autom..

[44] Taheri H., Qiao B., Ghaeminezhad N. (2015). Kinematic model of a four mecanum wheeled mobile robot. Int. J. Comput. Appl..

[45] Becker F., Bondarev O., Zeidis I., Zimmermann K., Abdelrahman M., Adamov B. (2014). An approach to the kinematics and dynamics of a four-wheel Mecanum vehicle. Sci. J. IFToMM Probl. Mech..

[46] Abdelrahman M., Zeidis I., Bondarev O., Adamov B., Becker F., Zimmermann K. A description of the dynamics of a four wheel mecanum mobile system as a basis for a platform concept for special purpose vehicles for disabled persons. Proceedings of the 58th Ilmenau Scientific Colloquium.

[47] Gao X., Wang Y., Zhou D., Kikuchi K. (2009). Floor-cleaning robot using omni-directional wheels. Ind. Robot Int. J..

[48] Li Y., Dai S., Shi Y., Zhao L., Ding M. (2019). Navigation simulation of a Mecanum wheel mobile robot based on an improved A* algorithm in Unity3D. Sensors.

